# Stroke Action Plan for Europe 2018–2030 (SAP-E): mid-term review and update

**DOI:** 10.1093/esj/aakaf026

**Published:** 2026-01-19

**Authors:** Hanne Christensen, Francesca Romana Pezzella, Melinda Berg Roaldsen, Aleš Tomek, Arlene Wilkie, Louisa Christensen, Martin Dichgans, Avril Drummond, Tiina Laatikainen, Carlos A Molina, Katharina S Sunnerhagen, Danilo Toni, Sonia Abilleira, Diana Aguiar de Sousa, Anita Arsovska, Heinrich Audebert, Jelena Bartolovic, Yannick Béjot, Geert Jan Biessels, Juliet Bouverie, Hrvoje Budincevic, Barbara Casolla, Hugues Chabriat, Marina Charalambous, Jesse Dawson, Stephanie Debette, Frank-Erik de Leeuw, Adam Denes, Marina Diomedi, Diederik Dippel, Ulrich Dirnagl, Urs Fischer, Yuriy Flomin, Ana Catarina Fonseca, Birgitte Forchammer, Anne Forster, Giovanni Frisullo, Miquel Galofre, Zuzana Gdovinová, Christoph Gumbinger, Joseph Harbison, Richard Hobbs, Dalius Jatuzis, Hrvoje Jurlina, Mira Katan, Lisa Kidd, Stefan Kiechl, Janika Kõrv, Christina Kruuse, Wilfried Lang, Arthur Liesz, Svetlana Lorenzano, Andreas Luft, Grethe Lunde, Chris Macey, Hugh Stephan Markus, Gillian Mead, Patrik Michel, Serefnur Ozturk, Maurizio Paciaroni, Aleksandra Pavlovic, Carina U Persson, Terence J Quinn, Peter Rothwell, Luca Saba, Paola Santalucia, Gustavo Santo, Claus Simonsen, Thorsten Steiner, Katarzyna Stolarz-Skrzypek, Cristina Tiu, Alexander Tsiskaridze, Georgios Tsivgoulis, Jaakko Tuomilehto, Teresa Ullberg, Paolo Ursillo, Antonella Urso, Mia van Euler, Margus Viigimaa, Denis Vivien, Markus Wagner, Marion Walker, Alastair Webb, Diana Wong Ramos, Mauro Zampolini, Marialuisa Zedde, Gary Ford, Peter Kelly, Robert Mikulik, Bo Norrving, Hariklia Proios, Simona Sacco, Else Sandset, Joanna Wardlaw, Aleksandras Vilionskis, Valeria Caso

**Affiliations:** Department of Neurology, Copenhagen University Hospital, Bispebjerg, Copenhagen, Denmark; Department of Neuroscience, San Camillo-Forlanini Hospital, Rome, Italy; Clinical Research Department, University Hospital of North Norway, Tromsø, Norway; Department of Clinical Medicine, UiT, The Arctic University of Norway, Tromsø, Norway; Neurology Department, Second Medical Faculty of Charles University and University Hospital Motol, Prague, Czech Republic; Stroke Alliance for Europe, London, United Kingdom; Department of Neurology, Copenhagen University Hospital, Bispebjerg, Copenhagen, Denmark; LMU Clinic, Institute for Stroke and Dementia Research, Munich, Germany; School of Health Sciences, University of Nottingham, Nottingham, United Kingdom; Institute of Public Health and Clinical Nutrition, University of Eastern Finland, Kuopio, Finland; Hospital Vall d’Hebron, Barcelona, Catalonia; Department of Rehabilitation Medicine, University of Gothenburg, Gothenburg, Sweden; University La Sapienza, Rome, Italy; Ministry of Health, Stroke Programme for Catalonia, Fundació TIC Salut Social, Bracelona, Catalonia; Lisbon Central University Hospital, ULS São José, Lisbon, Portugal; Faculdade de Medicina, Universidade de Lisboa, Gunbenkian Institute for Molecular Medicine, Lisbon, Portugal; Stroke Center, University Clinic of Neurology, University “Ss. Cyril and Methodius,” Faculty of Medicine, Skopje, North Macedonia; Department of Neurology and Center for Stroke Research, Charité Universitaetsmedizin, Berlin, Germany; University Hospital Sveti Duh, Zagreb, Croatia; Department of Neurology, Dijon University Hospital, Dijon, France; Department of Neurology, UMC Utrecht, Utrecht, The Netherlands; Stroke Association, London, United Kingdom; Department of Neurology, Sveti Duh University Hospital, Zagreb, Croatia; Department of Neurology, Université Cote d'Azur UR2CA-URRIS, CHU Hôpital Pasteur 2, Nice, France; Department of Neurology, CNVT (APHP) and INSERM, University of Paris Cité, Paris, France; Department of Rehabilitation Sciences, Cyprus University of Technology, Limassol, Cyprus; School of Cardiovascular and Metabolic Health College of Medical, Veterinary & Life Sciences, University of Glasgow, Glasgow, United Kingdom; Department of Epidemiology and Neurology, University of Bordeaux, Bordeaux University Hospital, Inserm, Bordeaux, France; Department of Neurology, Research Institute for Medical Innovation, Radboudumc, Nijmegen, The Netherlands; HUN-REN Institute of Experimental Medicine, Budapest, Hungary; University Hospital of Rome Tor Vergata, Rome, Italy; Department of Neurology, Erasmus MC Stroke Center Erasmus MC University Medical Center, Rotterdam, The Netherlands; Department of Experimental Neurology, Charite Universitätsmedizin Berlin and Berlin Institute of Health, Berlin, Germany; Department of Neurology, University Hospital Bern and University of Bern, Bern, Switzerland; Department of Neurology, Shupyk National University of Healthcare of Ukraine and Medical Center “Universal Clinic ‘Oberig’”, Kyiv, Ukraine; Department of Neurology, Hospital de Santa Maria, Faculdade de Medicina, Universidade de Lisboa, Lisboa, Portugal; Danish Stroke Association, Copenhagen, Denmark; University of Leeds, Leeds, United Kingdom; Department of Neurology, Fondazione Policlinico Agostino Gemelli, IRCCS - Department of Neuroscience, Catholic University of the Sacred Heart, Rome, Italy; Faculty of Medicine and Health, Department of Neurology and Rehabilitation, Örebro University, Örebro, Sweden; Department of Neurology, P.J. Safarik University Kosice, Faculty of Medince and University Hospital L. Pasteur Kosice, Slovakia; Department of Neurology, Heidelberg University Hospital, Heidelberg, Germany; Department of Medical Gerontology, Trinity College Dublin and Irish National Audit of Stroke, Dublin, Ireland; University of Oxford, Oxford, United Kingdom; Institute of Clinical Medicine, Faculty of Medicine, Vilnius University, Vilnius, Lithuania; Stroke Survivor, Stroke Alliance for Europe, Faculty of Family medicine, Zagreb Medical School, Zagreb, Croatia; Department of Neurology, University Hospital of Basel, Basel, Switzerland; Department of Nursing, School of Health & Life Sciences, Glasgow Caledonian University, Glasgow, United Kingdom; Department of Neurology, Medical University of Innsbruck, , VASCage, Innsbruck, Austria; Department of Neurology and Neurosurgery, University of Tartu, Tartu, Estonia; Department of Brain and Spinal Cord Injury, Neuroscience Center, Copenhagen University Hospital—Rigshospitalet, Copenhagen, Denmark; Department Neurology, Neurovascular Research Unit, University Hospital - Herlev Gentofte, Herlev, Denmark; Medical Faculty, Sigmund Freud Private University, Vienna, Austria; Department of Neurology, LMU Hospital, Munich, Germany; Department of Human Neurosciences, Sapienza, University of Rome, Rome, Italy; Cereneo Center for Neurology and Rehabilitation, University Hospital Zurich, Vitznau, Switzerland; Stroke Survivor, Stroke Alliance for Europe, Norway; Stroke Alliance for Europe, London, United Kingdom; University of Cambridge, Cambridge, United Kingdom; Stroke and Elderly Care Medicine, University of Edinburgh and NHS Lothian, Edinburgh, United Kingdom; Department of Neurology Service, Lausanne University Hospital and University of Lausanne, Lausanne, Switzerland; Department of Neurology, Selcuk University Faculty of Medicine, Konya, Turkey; Department of Neuroscience and Rehabilitation, University of Ferrara, Ferrara, Italy; Department of Neurology, Faculty of Special Education and Rehabilitation, University of Belgrade, Belgrade, Serbia; Department of Neuroscience and Physiology, Rehabilitation Medicine, Institute of Neuroscience and Physiology, Sahlgrenska Academy, University of Gothenburg, Gothenburg, Sweden; Department of Medicine, University of Gothenburg and Department of Medicine, Geriatrics and Emergency Medicine, Centre for Lifestyle Intervention, Sahlgrenska University Hospital/Östra, Gothenburg, Region Västra Götaland, Sweden; Department of Occupational Therapy and Physiotherapy, Sahlgrenska University Hospital/Östra, Gothenburg, Region Västra Götaland, Sweden; Department of Geriatric Medicine, University of Glasgow, Glasgow, United Kingdom; Department of Neurology, University of Oxford, Oxford, United Kingdom; Department of Radiology, AOU Cagliari, Cagliari, Italy; Department of Neurology, Staff Direzione Strategica, AOSGA, Roma, Italy; Department of Neurology, Hospitais da Universidade de Coimbra, Unidade Local de Saúde, Coimbra, Portugal; Department of Neurology, Aarhus University Hospital, Aarhus, Denmark; Department of Neurology, Varisano Krankenhaus Frankfurt Höchst, Frankfurt, Germany; Department of Neurology, Heidelberg University Hospital, Heidelberg, Germany; First Department of Cardiology and Hypertension, Jagiellonian University Medical College, Krakow, Poland; Department of Clinical Neurosciences, University of Medicine and Pharmacy “Carol Davila”, Bucharest, Romania; Ivane Javakhishvili Tbilisi State University, Pineo Medical Ecosystem, Tbilisi, Georgia; Second Department of Neurology, “Attikon” University Hospital, School of Medicine, National and Kapodistrian University of Athens, Athens, Greece; Department of Public Health, University of Helsinki, Helsinki, Finland; Department of Neurology, Skåne University Hospital in Malmö/Lund, Malmö, Sweden; Department of Clinical Sciences, Neurology, Lund University, Lund, Sweden; Department of Hygiene and Public Health, Agenas—Italian Agency for Regional Health Services, Rome, Italy; Department of Health Economics, Health Department of the Lazio Region, Rome, Italy; Clinical Research Department, University Hospital of Northern Norway, Tromsø, Norway; Department of Clinical Medicine, UiT, The Arctic University of Norway, Tromsø, Norway; Center of Cardiology, Tallinn University of Technology, North Estonia Medical Centre, Tallinn, Estonia; Department of Neurobiology, Centre Hospitalier Universitaire Caen Normandie, Caen, France; German Stroke Foundation, Gladbeck, Germany; Department of Stroke Rehabilitation, University of Nottingham, Nottingham, United Kingdom; Department of Stroke Medicine, Department of Brain Sciences, Imperial College London, London, United Kingdom; Portugal AVC—União de Sobreviventes, Familiares e Amigos, Lisbon, Portugal; Department of Neurology and Physical and Rehabilitation Medicine, Department of Rehabilitation, USLUMBRIA2, Foligno, Italy; Stroke Unit, Azienda Unità Sanitaria Locale-IRCCS di Reggio Emilia, Reggio Emilia, Italy; Radcliffe Department of Medicine, University of Oxford, Oxford, United Kingdom; Department of Stroke Service, Mater University Hospital, Dublin, Ireland; Department of Neurology, University College Dublin, Dublin, Ireland; Department of HRB Stroke, Clinical Trials Network Ireland, Dublin, Ireland; Department of Mater Misericordiae, University Hospital, Dublin, Ireland; School of Medicine, University College Dublin, Dublin, Ireland; Health Research Board, Stroke Clinical Trials, Network Ireland, Dublin, Ireland; Department of Neurology, International Clinical Research Center, St Anne’s University Hospital, Masaryk University Brno, Brno, Czech Republic; Neurology Department, Tomas Bata Hospital, Zlín, Czech Republic; Department of Neurology, Lund University Department of Clinical Sciences Lund, Skåne University Hospital, Lund, Sweden; Department of Neurocognitive Disorders and Rehabilitation, Department of Educational and Social Policy, University of Macedonia, Thessaloniki, Greece; Department of Biotechnological and Applied Clinical Sciences, University of L'Aquila, L'Aquila, Italy; Department of Neurology, Oslo University Hospital, Oslo, Norway; Centre for Clinical Brain Sciences, University of Edinburgh, UK and UK Dementia Research Institute Centre, University of Edinburgh, Edinburgh, United Kingdom; Stroke Center, Republican Vilnius University hospital, Vilnius, Lithuania; Department of Emergency and Cardiovascular Medicine, Santa Maria Della Misericordia, Perugia, Italy

**Keywords:** epidemiology, Europe, prevention, quality assurance, strategic planning, stroke, stroke services, treaties, treatment

## Abstract

**Objectives:**

Implementation of the Stroke Action Plan for Europe (2018–2030) (SAP-E) was initiated in 2019. It is now updated at mid-term to reflect and respond to challenges for stroke care in Europe in 2025.

**Methods:**

The SAP-E covers the entire chain of stroke care. The sections (state of the art, current status and targets) were developed by working groups and finalised based on inputs from the Interim Review Committee and an open online meeting. Targets for 2030 were updated to reflect current knowledge, to prioritise and to increase accountability.

**Results:**

All sections have been updated based on the newest evidence to reflect the state of the art and current status in 2025.

**Conclusion:**

Stroke remains a significant health issue in Europe, with notable incidence and inequities in access to care. Key interventions are strongly evidence-based, cost-effective and supported by World Health Organization and European Union recommendations. Despite improvements, gaps remain across the care pathway but particularly in terms of access to stroke units, rehabilitation and follow-up. To control and reduce the burden of stroke, the main action points are: (1) national stroke plans, which encompass the entire chain of care and are reflected in reimbursement systems, (2) quality and outcome control, where impact is measured at both individual and health care system level, (3) robust and resilient health care organisation covering the entire chain of care that promotes equal access to sustainable, timely and evidence-based stroke care and (4) effective national strategies to promote and facilitate a healthy lifestyle and risk factor control.

## Graphical Abstract

**Graphical Abstract:**
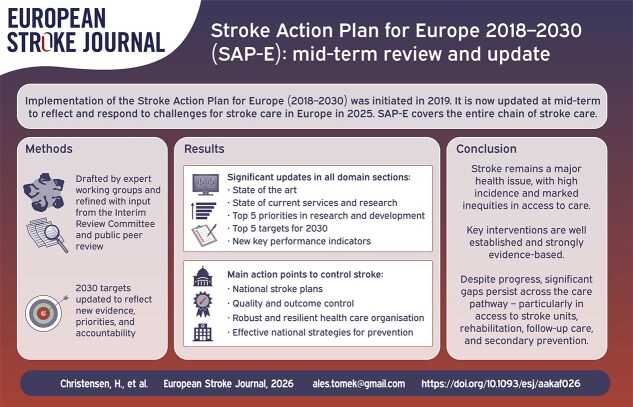

## Introduction

The absolute number of strokes in the World Health Organization (WHO) European region (EU-53) is still increasing, and there are considerable discrepancies in incidence, prevalence, mortality and disability-adjusted life-years between individual countries, with consistently lower rates in the European Union (EU-28) compared with EU-53.^1^ Several initiatives have been launched to halt or decrease the global burden of non-communicable diseases (NCDs), including stroke. Prevention and treatment of stroke—including primary prevention, thrombolysis, stroke unit care and secondary prevention—are now listed as “NCD Best Buys” by the WHO, underlining the cost-effectiveness of these interventions.^2^ Rehabilitation is also identified by the WHO as an essential part of universal health coverage.^3^ Consequently, all steps in the chain of stroke treatment, except life after stroke, are included in recent WHO recommendations, documenting the importance of the interventions and the strength of the evidence. Furthermore, the WHO Global NCD Action Plan 2013–2020 recognised the primary role and responsibility of governments in responding to NCDs and the role of international cooperation in supporting national efforts.^4^

In the larger perspective of brain health, it is important to note that modifying cardiovascular risk factors not only reduces the risk of stroke (and other cardiovascular diseases) but also maintains brain health and prevents dementia later in life,^5^ strongly linking cardiovascular risk reduction to brain health and brain health initiatives. In Europe, the EU NCD initiative “Healthier Together” (2022) prioritises developing national stroke plans that encompass the entire care chain.^6^ It includes its own set of “best practices,” which mirror the WHO Best Buys and other initiatives for stroke.

The Stroke Action Plan for Europe (SAP-E) 2018–2030 was developed by the ESO and Stroke Alliance for Europe and complements the above initiatives.^7^ Implementation of the SAP-E was initiated by establishing an implementation committee in 2019, which set up a strategic network and plan to meet the targets of this SAP-E.^8^ The COVID-19 pandemic significantly hampered initial progress, and at first, the programme was purely online.^9^ The SAP-E is anchored in its network of national coordinators (representatives from national scientific societies and stroke support organisations [SSOs]), who link the European-level initiative to national governments, healthcare professionals and patient organisations. Establishing national stroke plans is the highest priority for SAP-E to improve the lives of all people affected by stroke, recognising the primary role of governments in response to NCDs.^10^

To actively facilitate improvement of stroke care in Europe, key performance indicators (KPIs) 2030 were defined^11^ in collaboration with the national coordinators. The Stroke Service Tracker (SST) was established in 2020, and annual European aggregated summary data have been collected since then. The KPIs, as well as essential stroke variables, have been published.^12^

Using these tools, national coordinators have approached their national governments to secure commitment to implementing the SAP-E in their countries. This commitment was in the form of a Declaration and has been signed by 13 European countries,^13^ and 15 countries had a national stroke plan in 2022 compared to 8 in 2020.

As the original plan approached its mid-term, an update of the SAP-E was required to update the state of the art on stroke care, describe the present state of stroke care and increase accountability of the plan.

Four overarching targets remain the primary goal of the SAP-E. Only the first has been modified to increase operationality by including age-standardised incidence and increasing the target to 15%:

1. to decrease the age-standardised incidence of stroke by 15% from 2020 to 2030
2. to treat 90% or more of patients with acute stroke in Europe in a dedicated stroke unit as the first level of care
3. to have national plans for stroke encompassing the entire chain of care from primary prevention to life after stroke
4. to fully implement national strategies for multisector public health interventions to promote and facilitate a healthy lifestyle and reduce environmental (including air pollution), socioeconomic and educational factors that increase the risk of stroke.

## Methods

The work was planned and led by the leadership of SAP-E. The review and writing process followed the process previously used, which is described in the Helsingborg Declaration and first Action Plan for Stroke in Europe.^7^ In short, working groups for 8 domains (Primary Prevention, Organisation of Acute Stroke Services, Management of Acute Stroke, Secondary Prevention and Follow-up, Rehabilitation, Life After Stroke, Evaluation of Outcomes and Quality Improvement and Translational Stroke Research) were established based on the same stroke experts (if still active in the field) and the addition of new experts taking into account geographic origin, age and sex. With the overall purpose of ensuring representativeness, patient representatives were included in all groups. An Interim Review Oversight Committee, including patient representatives, was established to ensure transparency in the work. The groups reviewed the previous action plan with a focus on the need for updating based on new knowledge, the current state of services and accountability of the targets. The focus was on prevalent presentations in adult stroke. The existing SAP-E KPIs were integrated into the listed targets, and new KPIs were developed, when relevant (Table 1). The current state of services was supported by SST data, when available.^14^

**Table 1:** The list of Key Performance Indicators of the Stroke Action Plan for Europe, their definitions and benchmarks.

**KPI**	**Definition**	**Benchmark**
KPI 1	A national stroke plan defining pathways, care and support after stroke, including pre-hospital phase, hospital stay, discharge and transition, and follow-up.	Implemented
KPI 2	At least 1 individual from the respective SSO (if existent) will be involved and supported, in an equal way, during the development of each country’s national stroke plan or stroke-related guideline.	Implemented
KPI 3	A national strategy for multi-sectorial public health interventions that promote and facilitate a healthy lifestyle and risk factor control has been implemented. A national brain health plan including stroke-specific health factors across the life course has been developed. Nationwide pathways for opportunistic screening for key risk factors, including hypertension, dyslipidaemia, hyperglycaemia and atrial fibrillation. Has been implemented.	Implemented
KPI 4	Establishment of national- and regional-level systems for assessing and accrediting stroke clinical services, providing peer support for quality improvement, and making audit data available to the public**.**	Implemented
KPI 5	All stroke units and other stroke services independent of sector undergo quality auditing continuously or at regular intervals: Hospitals Other services.	Implemented
KPI 6	Access to stroke unit care for patients with acute stroke: Percentage admitted to stroke unit care Percentage admitted to stroke unit care within 24 h of arrival.	90% 90%
KPI 7	Recanalisation treatment provided for patients with ischaemic stroke: Percentage of patients treated with IVT Percentage of patients treated with MT Median door-to-needle times (IVT) Median door-to-groin times (MT).	20% 7.5% <30 min <60 min
KPI 8	Stroke units with access to: CT/MRI, vascular imaging, ECG, long-term ECG-monitoring, cardiac echo (TTE, TOE), dysphagia screening and blood tests during stroke unit admission.	90%
KPI 9	Access to early stroke unit rehabilitation including ESD. Percentage of stroke units with access to early stroke unit rehabilitation Percentage of stroke units with access to ESD.	90%
KPI 10	Access to basic secondary prevention, including antithrombotics, antihypertensives, statins and lifestyle advice.	90%
KPI 11	A binding, personalised, documented rehabilitation and sector transition plan is provided at the time of discharge.	70%
KPI 12	Follow-up at 3–6 months after the stroke incident, including a post-stroke checklist, functional assessment and referral for relevant interventions: Follow-up at 3–6 months Use of post-stroke checklist, functional assessment and referral for relevant interventions at follow-up.	Implemented
KPI 13	Percentage of patients in whom short-term mortality (30 days) after stroke is monitored and at acceptable levels for: ischaemic stroke ICH all stroke SAH	<10% <30% <15% <25%

Abbreviations: ESD = early supported discharge; SAH = subarachnoid haemorrhage.

A public livestreamed and recorded meeting was held on 27 August 2024, with 94 registered participants. All sections were systematically discussed, and working groups reviewed the sections after the meeting and considered feedback given during the meeting. The final version was subsequently reviewed by the Interim Review Oversight Committee and all working groups before submission for publication. The process was supported by the Head Office of the ESO, guaranteeing independence from other stakeholders.

### Primary prevention

Primary prevention of stroke—that is, prevention of a first stroke—is essential for overall brain and cardiovascular health. The WHO’s strategy to optimise brain health throughout the life course, emphasising stroke-specific risk factors, is therefore crucial.^15^ Our SAP-E aligns with other key preventive initiatives, including brain health strategies from the American Heart Association, American Stroke Association and European Academy of Neurology.^16^^,^^17^ Structural interventions include enacting legislative changes, implementing taxation, involving the food industry, imposing advertising and sales restrictions and employing various fiscal policies. The goal is to reduce tobacco and nicotine product use notably, curb harmful alcohol consumption, promote healthier dietary habits and discourage sedentary lifestyles. To identify risk factors in individuals before they lead to stroke, we need to implement pathways for nationwide opportunistic screening strategies for key risk factors, including hypertension, dyslipidaemia, hyperglycaemia and atrial fibrillation (AF).

#### State of the art

One of the 4 overarching targets of SAP-E is to reduce the age- and sex-standardised incidence of stroke by more than 15% by 2030. Given the rapidly ageing population in Europe, primary prevention at an early stage is increasingly critical to avert the escalating disease burden. Most of the stroke risk—spanning age, sex and ethnicity—is attributed to a few key modifiable factors: smoking, hypertension, dyslipidaemia, unhealthy diet, excessive alcohol intake, physical inactivity, obesity, diabetes and cardiac diseases (including AF).^18^ Recent evidence adds insufficient sleep, substance abuse, e-cigarettes, psychosocial factors and environmental factors such as air quality as stroke risk factors.^19^

#### Public health interventions

Public health interventions promoting a healthy lifestyle and targeting highly prevalent risk factors that do not require pharmacological intervention should be deployed on multiple fronts. These interventions may encompass legislative changes, taxation and other fiscal policies, as well as reformulation and labelling of food. As an example, reducing salt intake at the population level and effective control of hypertension are crucial for preventing strokes. This preventive approach should be implemented across individual, community and population levels. In addition, media campaigns and educational and preventive measures in schools, workplaces and communities play a pivotal role in this comprehensive strategy.

Both population-wide and high-risk strategies are needed for efficient reduction of the incidence of strokes.^20^ A significant proportion of strokes occur in individuals with low- or intermediate-risk profiles.^21^ In stroke prevention, it is important to address diverse demographics, including younger individuals, those with low socioeconomic status, and people from various genetic and ethnic backgrounds.^22^

Given the substantial prevalence of potent stroke risk factors, it is advisable to implement comprehensive prevention strategies aimed at the general population. Systematic screening improves the identification of risk factors, yet uncertainties persist regarding the beneficial influence of screening on clinical outcomes.^23^^,^^24^ Opportunistic screening and screening of high-risk populations for atherosclerotic cardiovascular disease based on individual risk assessment tables—such as the Systematic COronary Risk Evaluation^25^ or Stroke Riskometer,^26^ which use risk factors like blood pressure, blood glucose and lipids—enhances detection rates and is recommended.^27^^,^^28^ Opportunistic screening should also be considered in patients with diagnosed covert infarcts and covert cerebral small vessel disease (SVD) who do not exhibit overt neurological symptoms. These patients typically have a higher prevalence of traditional cardiovascular risk factors and events.^29^

#### Risk factor modification

Ample evidence indicates that treating cardiovascular risk factors reduces stroke risk.^30^ However, target levels in primary prevention are less strict than in secondary prevention, vary with comorbidities such as diabetes and active smoking and may differ by sex.^27^ Further details are given in Table 2.

**Table 2:** Key policy and healthcare recommendations for stroke prevention targeting major modifiable risk factors at the population and healthcare system level.

**Risk factor**	**Recommendations for action**
Smoking	Encourage national governments to impose annual above-inflation rate tax increases on tobacco products. Restrict sales of tobacco products and ban all tobacco advertising and sponsorship including electronic cigarettes.
Electronic cigarettes	Support measures to ban the sale of e-cigarettes to minors, all advertising and sponsorship and all flavours apart from tobacco and disposable vapes, along with plain packaging of e-cigarettes and specific taxes on e-liquids.
Alcohol	Encourage national governments to enact and enforce restrictions on the physical availability of retailed alcohol (via reduced hours of sale). Increase excise taxes on alcoholic beverages and ban advertising across multiple types of media.
Diet	Adopt national policies to reduce population salt/sodium consumption, limit saturated fatty acids and red meat and eliminate trans fatty acids in the food supply. Policies could include health-based taxing and pricing support for healthy food products.
Obesity	Halt the rise of obesity in populations as a crucial target in stroke prevention.
Physical activity	Address the importance of sufficient physical activity (at least 150 min/week) in stroke prevention.
Elevated blood pressure	European national medical professional societies should commit to and adhere to recommendations for managing blood pressure in patients with hypertension.
Dyslipidaemia	European national medical professional societies should commit to and adhere to recommendations for managing dyslipidaemia.
Psychosocial factors	Stress symptoms and psychosocial stressors modify CVD risk.^27^ Assessment of these stressors, including depression, anxiety and insomnia, should be considered.
Diabetes	Actively employ non-laboratory risk scores for screening T2DM risk (SCORE2-Diabetes). Individuals with elevated scores should undergo assessments for glycemia and CVD risk factors and ensure optimal prevention.
Atrial fibrillation	European national medical professionals should follow recommendations concerning screening for atrial fibrillation and prevention of embolic events in patients with atrial fibrillation.
Kidney diseases	Emphasise the availability of therapeutic agents that effectively reduce albuminuria and mitigate CVD risk. Healthcare providers should consider incorporating these agents into comprehensive care plans.
Air pollution	European states should commit to WHO air quality guidelines,^31^ which recommend levels and interim targets for particulate matter and other common air pollutants, nitrogen dioxide (NO_2_), sulphur dioxide (SO_2_) and ozone (O_3_) deriving from outside as well as household air pollution.

#### State of current services

**Figure 1:**
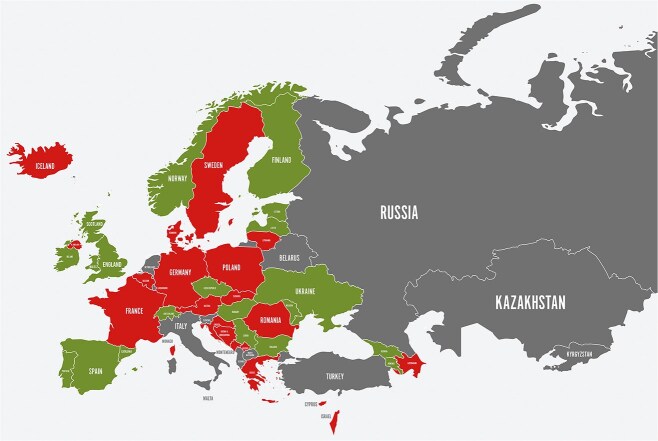
Countries are categorized according to color as follows: Green (Norway, Finland, United Kingdom (England, Scotland, Wales), Ireland, Spain, Portugal, Ukraine, Estonia, Latvia, Bulgaria, Armenia, Azerbaijan); Red (Iceland, Sweden, Denmark, Germany, Poland, France, Romania, Greece, Cyprus, Israel, Georgia, Lithuania, Belgium, Netherlands, Austria, Czech Republic, Slovakia, Hungary, Slovenia, Croatia, Bosnia and Herzegovina, Serbia, North Macedonia, Albania); Grey (Russia, Belarus, Italy, Switzerland, Turkey, Kazakhstan, Montenegro, Kosovo, Malta, Moldova, Kyrgyzstan).

Effective population-wide and high-risk prevention strategies differ throughout Europe, emphasising the importance of applying both approaches. Moreover, modifiable stroke risk factors and levels of awareness vary widely across European populations.^1^ According to SST data established by SAP-E, 20 countries of SAP-E implemented a strategy for interventions promoting a healthy lifestyle and risk factor control in 2022 (Figure 1) compared with 15 in 2021 and 11 in 2020.^31^

Despite the potential to save lives and reduce healthcare costs, the WHO’s recommended “NCD Best Buys” addressing tobacco, alcohol, diet and physical activity are inadequately implemented. An updated list of “NCD Best Buys” in 2023 provides more policy options and cost-effective interventions for governments to prioritise investments.^32^ While all European countries have ratified the WHO Framework Convention on Tobacco Control, implementation varies.^33^ A notable disparity exists between guideline-recommended risk-factor control and actual stroke prevention in the real world. Even with widespread access and healthcare coverage, a study found that 80% of people diagnosed with ischaemic stroke had at least 1 untreated or inadequately treated medical risk factor such as hypertension, hyperlipidaemia or AF.^34^

#### Research and development: top 5 priorities

1. Research on population strategies of stroke prevention at a governmental level and how to measure their efficacy—health economic approach and sustainability of those programmes.
2. Evidence on precision lifestyle medicine and precision medicine in preventing stroke.
3. Evidence to assess the benefits and potential harms of screening for stroke and stroke subtypes/aetiologies and cardiovascular disease risk factors in diverse populations, considering various approaches such as systematic and opportunistic screening.
4. Evidence on the effectiveness of digital health approaches in improving adherence with primary prevention interventions and their outcomes in stroke prevention.
5. Research on psychosocial factors and mental health concerning the risk and outcome of stroke.

#### Targets for 2030: top 5 priorities

1. Reducing the age- and sex-standardised incidence of stroke by more than 15% by 2030 compared with 2018 (this is updated from the previous target to reduce total number of strokes by 10%).
2. Fully implementing national strategies for multi-sectorial public health interventions promoting and facilitating a healthy lifestyle and risk factor control (KPI 3a).
3. Having key stroke risk factors—hypertension, dyslipidaemia, AF and hyperglycaemia—detected to the highest proportion and having people with high risk factors controlled, aiming at 80% of persons in target levels.
4. Implementing plans to promote brain health plans, including a focus on stroke-specific risk factors across the life course (KPI 3b—new)
5. Implementing pathways for nationwide opportunistic screening strategies for key risk factors, including hypertension, dyslipidaemia, hyperglycaemia and AF (KPI 3c—new).

### Organisation of acute stroke services

Organisation of stroke services is crucial to provide optimal treatment at every stage of care—from prevention to acute treatment to long-term care. Although specific stroke services are present in most European countries, there is significant variability in the practical application of treatment guidelines; adherence to quality indicators^35^^,^^36^; and definitions, requirements and use of terms. To be pragmatic, we use the descriptive terms “organised stroke unit care” and “acute stroke services.”

#### State of the art

Stroke awareness programmes on recognising stroke signs for the general public positively influence fast admission to acute stroke treatments but require regular repetition to maintain long-term effectiveness.^37–42^ Organisation of stroke care—from the pre-hospital phase to life after stroke—is key, as acute stroke treatment is very time sensitive, while later interventions, including rehabilitation and follow-up, need to be made available to many patients. Organisation of care should follow a defined national (or regional) stroke pathway and be based on a national stroke plan that covers the entire patient pathway.

Adequate training of emergency medical services (EMSs) personnel and dispatchers and the use of validated pre-hospital stroke identification tools improve stroke recognition and transport time,^43^^,^^44^ and digital solutions supported by artificial intelligence have the potential to further improve patient assessment and interaction between prehospital and in-hospital stroke care teams.^45–48^ Pre-notification of patient arrival to a multidisciplinary stroke team leads to shorter delays and more rapid management.^49^^,^^50^

Patients with acute stroke must be delivered to a hospital that provides an acute stroke service—including intravenous thrombolysis (IVT)—based on local organisation and geography. These hospitals should ensure that patients with suspected stroke have rapid and continuous access to vascular brain imaging,^51^ allowing for work-up according to the individual patient’s needs and best evidence according to guidelines. In RCTs, there was no benefit of direct transportation to a mechanical thrombectomy (MT)–capable centre for patients with LVO, but harms were observed in the subgroup of patients with ICH, in whom bypassing the closest stroke centre may result in reduced chances of functional independence at 90 days.^52^ The acute interventions IVT and MT are used more frequently and with higher quality in high-volume centres.^53^

Different modes of overcoming geographical challenges in access to acute stroke care have been explored. Helicopter transportation may be useful in specific settings, including rural, remote and intermediate-density areas.^54–57^ The concept of delivering personnel and equipment to the patient via mobile stroke units (MSUs) compared with usual care has led to earlier treatment, a significant increase in excellent outcomes and a reduction in onset-to-treatment times for IVT in urban areas.^58–60^ In remote areas, telemedicine is feasible and leads to improved acute stroke treatment, facilitating patient triage, accommodating IVT delivery and orchestrating drip-and-ship models or on-site MT with a flying (or driving) intervention team.^61–69^

A critical element of every stroke care system is a network of organised stroke units with complete geographical coverage and sufficient capacity to treat all stroke patients. Admission to organised stroke unit care as the first level of care is crucial to prevent complications and initiate early prevention and rehabilitation. Treatment in dedicated stroke units reduces the risk of disability, institutional care and death, regardless of age, sex, initial stroke severity and stroke type.^70–73^

#### State of current services

Although significant progress has been made, there is still considerable inequality in the organisation of stroke care in Europe, as shown by recent ESO studies assessing delivery of stroke care.^74^^,^^75^ Detailed and current information about the organisation and results of stroke care—from acute care through rehabilitation and life after stroke—is still lacking in many countries. In most European countries, a national stroke society supports coordination of stroke services and fosters quality improvements in stroke care.^76^

**Figure 2:**
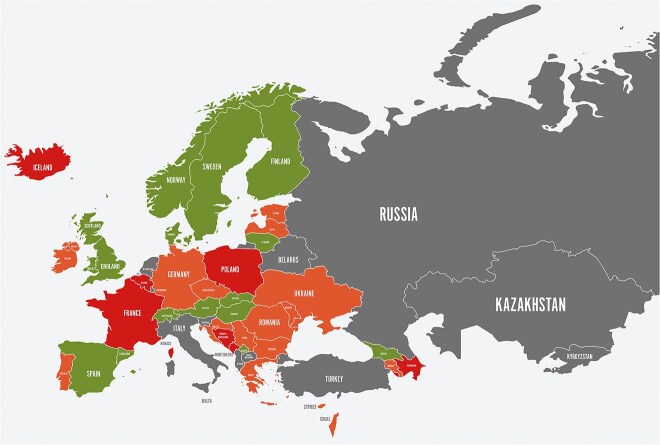
Countries are categorized according to color as follows: Green (Norway, Sweden, Finland, Denmark, United Kingdom (England, Scotland, Wales), Spain, Portugal, Austria, Switzerland, Slovenia, Hungary, Lithuania, Armenia, Georgia); Orange (Ireland, Germany, Ukraine, Romania, Bulgaria, Greece, Latvia, Estonia, Czech Republic, Slovakia, Croatia, Serbia, Bosnia and Herzegovina, Montenegro, North Macedonia, Albania);, Red (Iceland, France, Poland, Belgium, Netherlands, Cyprus, Israel, Azerbaijan); Gray (Russia, Belarus, Italy, Turkey, Kazakhstan, Malta, Moldova, Kosovo, Kyrgyzstan, Monaco).

According to 2022 SST data, 17 of 42 countries had established a national stroke plan, which is significant progress compared to 11 in 2021 (Figure 2) but still less than half of European countries.^77^ Close national collaborations between governments, SSOs and scientific societies must be built to set up comprehensive national stroke plans and ensure funding and implementation. In two-thirds of European countries, patient representatives are now involved in the development of national stroke plans and guidelines; however, patient involvement is still lacking in 14 countries.^31^ The target of access to stroke unit care within 24 h of onset as the first level of care in at least 90% of patients was only reached by 7 countries. Few countries monitor the timing of access to stroke unit care in spite of the time-sensitive nature of this intervention. Only 6 countries reported access for at least 75% of patients within 24 h.^31^

Most countries have an EMS system with regional organisation and written protocols for acute stroke. An increasing number of countries use pre-hospital notification of hyperacute stroke care,^76^ which is associated with better post-stroke outcomes. Training for EMS may improve pre-hospital stroke recognition and transport time; however, only limited information on the status of pre-hospital care in the various countries is available, and significant disparities still exist globally.^45^^,^^78^^,^^79^ Many countries do not have obligatory transport routes to the closest suitable stroke hospital.^80^

Although stroke symptoms and the importance of immediate action have repeatedly been communicated to the public, public education campaigns aimed at improving help-seeking behaviour by acute stroke patients until recently have achieved only limited effects. Awareness is still unsatisfactory among the general population, as only about 50% of the population would immediately call an ambulance. Future public education campaigns should focus on the need to call the EMS in case of stroke symptoms, even if daily activities do not seem to be severely impaired.^81^^,^^82^

The crucial impact of time in acute stroke—whether acute ischaemic stroke or ICH—has constantly been stressed,^83^ but fewer than 10% of stroke patients reach the hospital within 60 min of symptom onset. In many countries, the time interval between onset of symptoms and arrival at the emergency department (ED)—onset-to-door (OTD) time—has not changed significantly over time; however, data are very limited. In some countries, OTD times have worsened in recent years due to pressures on EMS services.

Several countries have built a nationwide network of hospitals with stroke units or stroke centres following written protocols. However, no complete information on definitions of stroke units and comprehensive stroke centres in these countries is available.

Only a few countries have established a continuous, permanent and sustainable quality improvement system with a predefined set of criteria that are regularly measured and compared with benchmarks to identify gaps and needs in stroke care. Further details are provided in Domain 7: “Evaluation of Outcomes and  Quality Improvement.”

Reimbursement structures for stroke care and, in general, costs related to each stage of stroke (primary prevention, acute stroke and post-stroke) are highly variable between European countries, leading to gaps in the quality of care in some countries. This variability may be due to differences in cost factors, which are considered the monetary value, the services offered by each health system and data access.^84^ Furthermore, diagnosis-related group (DRG)–based payment systems, which have become the main mechanism for reimbursement of acute inpatient care, can be inadequately low (or high) for highly variable, highly specialised and/or low volumes of care.^85^ In-depth research is needed to understand this issue better, as well as to understand the definition of a standard schedule for assessing costs of stroke to obtain comparable data and to understand the combination of DRG-based payments with other reimbursement mechanisms, such as outlier payment adjustment, exclusion of highly complex patients, various forms of additional budgets and fee-for-service payments.

OECD describes a health workforce crisis and reports that education and training remain the most important direct policy tool for building the health workforce. Insufficient availability of adequately trained staff—ie, interventionalist, neurologist, nurses and therapists—may be a limiting factor in providing and improving stroke care and education, recruitment and training must be included into planning of future stroke care.^86^

#### Research and development: top 5 priorities

1. What are the most relevant barriers to the implementation of evidence-based stroke care?
2. What is the health-economic impact of stroke and the return of investment in stroke care? Which are the most cost-effective concepts to improve organisation of stroke care in countries with limited resources?
3. What are the optimum numbers and ratios of stroke centres and stroke units per million population for municipal and rural areas?
4. What is the role of telemedicine systems for acute stroke, rehabilitation and long-term care?
5. What elements are needed to enable more effective participation in decision-making among patients and relatives?

#### Targets for 2030: top 5 priorities

1. Implementing a national stroke plan that defines pathways, care and support after a stroke, including pre-hospital phase, hospital stay, discharge and transition, follow-up and life after stroke. These pathways should involve the public and should be adaptable to regional circumstances to ensure equal access to stroke care, regardless of patient age, characteristics, region and time of hospitalisation (KPI 1).
2. Establishing a scientific stroke society and SSO in each country.
3. Having at least 1 individual from the respective SSO equally involved and supported during the development of each country’s national stroke plan and stroke-related guidelines (KPI 2).
4. Treating 90% or more of all patients with acute stroke in Europe in a stroke unit as the first level of care (KPI 6a).
5. Treating 90% or more of all patients with acute stroke in Europe in a stroke unit within 24 h after admission to hospital as the first level of care (KPI 6b).

### Management of acute stroke

#### State of the art

##### Ischaemic stroke

Acute stroke is a medical emergency. The benefit of recanalisation therapies in patients with acute ischaemic stroke is strongly time-dependent, with earlier intervention achieving better outcomes.^87^ Stroke care systems should, therefore, minimise the time to assessment and initiation of treatment.^88^^,^^89^ Pre-hospital stroke management and organisation of acute stroke care are covered in Domain 2: “Organisation of Acute Stroke Services.”

##### Hospital admission

All patients with suspected stroke should be admitted to hospital for assessment and included in the stroke network, with access to stroke expertise (see Domain 2: “Organisation of Acute Stroke  Services”). Patients should be admitted to a stroke unit in a hospital with a defined rapid pathway for acute stroke management and staff with expertise in acute stroke care; admission to an organised stroke unit should be the first level of care. To discriminate ischaemic and haemorrhagic stroke and exclude other structural causes of the patient’s symptoms, immediate brain imaging with non-contrast CT or (MRI: DWI, thick-/thin-section susceptibility-weighted imaging, FLAIR) should be performed in patients with ongoing symptoms.^90^ For patients arriving with an unknown time of onset within 6–24 h and potentially eligible for IVT or MT, MRI ± MRA ± MRI perfusion or CT + CTA + CTP imaging should be performed. Basic tests are presented in Table 3.

**Table 3:** Recommended diagnostic investigations in patients with suspected or confirmed stroke, stratified by stroke type and underlying aetiology. The table outlines core assessments performed in all patients.

**Stroke type**	**Aim**	**Investigation**
All	Ischaemic vs haemorrhagic	Admission CT ± CTA, or MRI ± MRA
	Neurological status	Stroke severity rating scale (eg, National Institutes of Health Stroke Scale)
	Vital measures	Blood pressure, weight/body mass index
	Blood tests and ECG	Lipids, glucose, HbA_1c_, coagulation, eGRF and electrolytes, full blood count, ECG
	Lifestyle risk factors	Targeted interview (smoking, alcohol, diet, physical activity and other lifestyle risk factors)
Ischaemic/TIA	Large artery stroke	Admission brain and vascular imaging (CTA, MRA, Doppler ultrasound)
	Small vessel stroke	Admission brain imaging
	Atrial fibrillation	ECG and prolonged rhythm monitoring
	Embolic stroke	Echocardiography (TTE/TOE) and consider other major embolic sources In patients with likely central embolism (no lacunar or large artery features, including non-stenosing plaques), cardiac echocardiography (TTE and TOE when indicated) and prolonged ECG monitoring should be performed. Right-to-left shunt can be screened using TCD^91^
	Dissection	Admission vascular imaging CTA, Doppler and/or MRI, with wall haematoma optimised.
ICH		Intracranial CTA; digital subtraction angiography if appropriate, MRI with GRE or SWI sequences. Consider additional imaging based on suspected aetiology (eg, blood-sensitive MRI, CVT protocol)^92^

Abbreviations: CT = computed tomography; CTA = computed tomography angiography; MRI = magnetic resonance imaging; MRA = magnetic resonance angiography; NIHSS = National Institutes of Health Stroke Scale; ECG = electrocardiogram; HbA1c = glycated haemoglobin; eGFR = estimated glomerular filtration rate; TTE = transthoracic echocardiography; TOE = transoesophageal echocardiography; TCD = transcranial Doppler ultrasound; GRE = gradient recalled echo; SWI = susceptibility-weighted imaging.

##### Intravenous thrombolysis

The earlier treatment with IVT is initiated, the greater the benefit, irrespective of age and stroke severity. Timely restoration of blood flow through IVT improves outcomes after stroke (number needed to treat [NNT] 5–9).^93^

IVT should be given within 4.5 h from symptom onset and in patients with unknown onset (wake-up patients) if there is a mismatch on the MRI.^94^ Special patient groups, including those with contraindications and those outside traditional time windows, may still benefit from these treatments under specific conditions based on individualised assessment.^93^ Tenecteplase (0.25 mg/kg, maximum 25 mg) is non-inferior to alteplase (0.9 mg/kg, maximum 90 mg) and is easier to administer as it is given as a bolus. In addition, recanalisation rates after MT in patients with LVO and time of symptom onset < 4.5 h were increased in patients treated with tenecteplase compared to those treated with alteplase.^93^ In patients with basilar artery occlusion (BAO), IVT is recommended for up to 24 h.^95^

##### Mechanical thrombectomy

In patients with anterior circulation LVO, MT is recommended within 6 h after stroke symptom onset (NNT 3). It should be offered for up to 24 h, depending on clinical or imaging evidence of salvageable brain tissue and collaterals in patients living independently.^90^ In patients with LVO, IVT is recommended before initiation of MT within the first 4.5 h after symptom onset. Large-core ischaemic stroke was excluded from early trials, but recent studies showed a significant benefit of MT in this subgroup of patients.^90^ For posterior circulation ischaemic stroke with BAO, MT showed an overall benefit up to 24 h for patients with at least 10 NIHSS points. However, MT is futile for patients with distal or medium vessel occlusion.^95–97^

##### Transient ischaemic attack

Prompt acute assessment, including imaging of extra- and intracranial vessels and relevant secondary prevention, should be provided in patients with TIA. In most cases, this will translate into the same work-up as performed in ischaemic stroke.^98^

##### Spontaneous ICH

Stroke unit care is at least as beneficial in patients with spontaneous ICH as in patients with ischaemic stroke and should thus be provided as soon as possible.^99^ In acute ICH, blood pressure should be lowered to systolic blood pressure at or below 140 mmHg as fast as possible and within 6 h. Blood-pressure lowering should be maintained for up to 7 days.^100^ In lobar ICH, early clot removal by minimally invasive surgery performed in centres with low complication rates improves outcomes in selected patients.^101^ Decompressive craniectomy in people with severe deep ICH may be considered^102^ to reduce mortality. In oral anticoagulant–related ICH, reversal agents have shown benefits in reversing iatrogenic coagulopathy and reducing haematoma expansion and so should be considered.^103^ Platelet suspension increased the risk of poor outcomes in patients with ICH on antiplatelets in 1 RCT.^104^

##### Subarachnoid haemorrhage

In patients with subarachnoid haemorrhage (SAH) caused by a rupture of an intracranial aneurysm, the primary goal is prevention and treatment of complications such as rebleeding, delayed cerebral ischaemia and hydrocephalus. These patients should be admitted to a unit with expertise in treatment of SAH. The risk of rebleeding can be reduced by occlusion of the aneurysm through coiling or clipping techniques; coiling is preferred in cases where both treatment options seem equally feasible. Nimodipine reduces the risk of delayed cerebral ischaemia and increases the chance of a favourable outcome.^105^ Early short-term treatment with tranexamic acid to reduce the risk of recurrent SAH before closure of the aneurysm has not shown benefit.^106^

##### Stroke unit care

All patients with ischaemic stroke or ICH benefit from special attention and organised care within a designated stroke unit to prevent poor outcomes (NNT 16).^107^ Organised stroke unit admission should be the first level of care, ideally immediately after arrival at the hospital. Systematic assessment components should include swallowing, temperature, nutrition, bowel and bladder function, skin breakdown, mobility, functional assessment and venous thromboembolism (VTE) prophylaxis. Swallowing tests should be performed in all patients as soon as possible,^108^ and VTE prevention should be provided in immobilised patients.^109^ Glycaemic control in non-diabetic patients^110^ and temperature control^111^ should not be delivered to improve outcomes after stroke. Immediate initiation of antiplatelet drugs is beneficial for preventing stroke recurrence in patients with ischaemic stroke not receiving IVT/MT; more information on initiation of secondary prevention is found in Domain 4: “Secondary Prevention and Follow-up.” Antiplatelet drugs should be started immediately in patients with ischaemic stroke; in case of IVT or MT, antiplatelet drugs should be initiated within 24 h.^112^ Stroke unit care should not be withheld from patients with uncertain rehabilitation potential.^113^

#### State of current services

**Figure 3:**
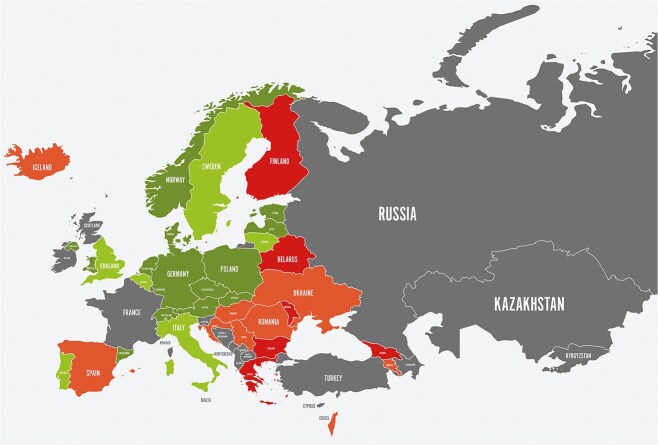
Countries are categorized according to color as follows: green—Norway, Sweden, Denmark, Germany, Poland, Italy, United Kingdom (England and Wales), Ireland, Estonia, Latvia, and Lithuania; orange—Iceland, Spain, Ukraine, Croatia, Montenegro, and Armenia; red—Finland, Belarus, Romania, Bulgaria, Greece, Cyprus, Israel, Georgia, and Azerbaijan; gray—France, Russia, Turkey, Kazakhstan, Kyrgyzstan, Serbia, Bosnia and Herzegovina, North Macedonia, Albania, Kosovo, Moldova, Malta, and Monaco.

Based on the 2022 SST data, IVT rates have increased yearly since 2020 in all regions of Europe. In total, 16 of 35 countries belonging to all regions of Europe with available national data report IVT rates above 15% (based on patients with ischaemic stroke), and 7 countries report rates above 20%. However, significant inequity remains, as 11 countries report rates at 9% or less and 4 countries even less than 5%. Seventeen countries were not able to report relevant national IVT data (Figure 3). The total number of IVT treatments reported by 33 countries in 2022 was 128,506 of 899334 ischaemic strokes. That is an IVT rate of 14.3% in Europe. IVT treatment is initiated earlier on a pan-European scale. In 2022, 10 countries reported national door-to-needle times shorter than 30 min.

In 2022, 5 countries provided MT to at least 10% of patients with ischaemic stroke; 17 countries were in the 5%–9% range. This development continues the significant increase in the use of MT observed in the 2021 SST dataset. However, in 12 countries, the rate of MT is below 5%. The total number of MT treatments reported by 33 countries in 2022 was 59,178 of 899,334 ischaemic strokes, ie, an MT rate of 6.6% in 35 European countries reporting data. Seven SAP-E countries reported reaching the target of median door-to-groin times below 60 min. Eleven countries reported 30-day case-fatality rates for ICH in the 2022 SST data. The reported range from countries with data based on national registry or national reimbursement data is 27% (Denmark) to 51% (Latvia). Eight countries reported 3-month mortality in the 2022 SST data. The lowest reported rate was 32% in Sweden, and the highest was 50% in Moldova.

#### Research and development: top 5 priorities

1. How can the speed, safety and effectiveness of reperfusion approaches (drugs or devices) be optimised in Europe?
2. Which pharmacological or other strategies will reduce the extent of irreversible brain damage in ischaemic stroke patients before recanalisation therapies are started?
3. Which strategies will improve outcomes in ischaemic stroke patients who are not eligible for reperfusion therapies or who do not recover after recanalisation?
4. Which treatment strategies will improve outcomes in patients with ICH: haemostatic and surgical approaches, prevention of secondary injury and intensive and tailored blood pressure management?
5. Which treatment strategies will further improve outcomes in patients with SAH by reducing brain injury?

#### Targets for 2030: top 5 priorities

1. Achieving national IVT rates above 20% of all patients with ischaemic stroke (KPI 7a).
2. Achieving national MT rates above 7.5% of all patients with ischaemic stroke (KPI 7b).
3. Median door-to-needle time <30 min in MT (KPI 7c) and median door-to-groin <60 min in IVT (KPI 7d—new).
4. First-month case-fatality rates <15% for all stroke patients (KPI 13a—new).
5. First-month case-fatality rates <10% after ischaemic stroke (KPI 13b—new), first-month case-fatality rates <30% for ICH (KPI 13c—new), and first-month case-fatality rates <25% for SAH (KPI 13d—new)

### Secondary prevention and follow-up

Early initiation of secondary prevention on arrival to hospital with a stroke is essential, as it can reduce recurrent stroke, cognitive decline, mood disturbances, fatigue, poor quality of life, other vascular events and associated functional impairment and mortality after stroke or TIA.

#### State of the art

Following diagnosis of ischaemic stroke or TIA, the aetiology (large artery disease, cardio-embolism, SVD and rare causes) should be identified, considering the possibility of multiple concurrent causes in the same individual (Table 3). This approach allows treatment with appropriate secondary preventive strategies (Table 4). Surgical or radiological procedures indicated for secondary prevention—such as carotid endarterectomy and stenting, closure of atrial septal defects and patent foramen ovale and atrial appendage occlusion—are highly operator dependent. Success rates depend on proper mentoring and training, as well as an adequate number of procedures being performed each year and should be monitored.

**Table 4:** Recommended secondary prevention interventions after stroke or transient ischaemic attack (TIA), stratified by stroke subtype and underlying mechanism.

**Stroke type**	**Intervention**
All	Primary prevention measures are applicable to secondary prevention; please refer to domain on primary prevention.
	Treat high blood pressure initially with a combination of 2 antihypertensives considering the potential risk of hypotension in some groups. Treatment target 130/80 mm Hg.
	Treatment of diabetes should include glucose-lowering agents with proven cardiovascular benefit to reduce the risk of future major adverse cardiovascular events.^114^^,^^115^
	Sex-specific differences exist related to menopause and andropause, eg, certain types of hormone-replacement therapy may increase the frequency and severity of stroke. Consider associations (hormonal birth control, migraine with aura, smoking).^116^
Ischaemic stroke/TIA	Antiplatelet therapy for non-cardioembolic stroke: aspirin and clopidogrel for 3 weeks in acute minor stroke and high-risk TIA, then monotherapy or aspirin and dipyridamole.
	Lipid lowering with a statin, ideally at maximum dose. In patients with TIA stroke and evidence of atherosclerosis treatment, target LDL cholesterol < 1.8 mmol/l, which may require addition of other agents, including ezetimibe or injectable therapies (PCSK9 inhibitors, inclisiran) to reach target. If statins cannot be tolerated, ezetimibe and bempedoic acid may be required to reach target LDL cholesterol level of <1.8 mmol/l.
Large artery disease	CEA or carotid stenting for symptomatic carotid stenosis (>50%) (NASCET score) when appropriate, as soon as the patient is stable and within 2 weeks.^117^
Cardioembolic stroke	In AF, DOAC is the first line of treatment; VKA is second line.^118^
	Device closure of PFO^119^ with a moderate to large shunt or an atrial septal aneurysm reduces recurrent events in patients < 60 years. LAAO can be considered in some patients with AF when anticoagulation is indicated but not tolerated. Collaboration in a neurocardiology setting is advisable for optimal patient selection for the procedures.
Lacunar stroke	Optimise control of blood pressure, blood glucose, lipids and antiplatelet therapy.
Other determined aetiology	Includes, among others, cervical artery dissection, cerebral venous thrombosis, recreational drugs and hereditary causes and requires specific investigation and treatment (in addition to treatment as defined in “All” above).
Intracerebral haemorrhage	Treating hypertension Modifying all other vascular risk factors and lifestyle (as above).
SAH	Stopping smoking, moderate alcohol intake and treating hypertension (as above). Non-invasive screening for first-degree family history if 2 or more first-degree relatives are affected.

Abbreviations: AF = atrial fibrillation; CEA = carotid endarterectomy; DOAC = direct oral anticoagulant; LDL = low-density lipoprotein cholesterol; LAAO = left atrial appendage occlusion; PCSK9 = proprotein convertase subtilisin/kexin type 9; PFO = patent foramen ovale; VKA = vitamin K antagonist.

A similar approach should be taken for diagnosis of ICH, which may be lobar (often due to cerebral amyloid angiopathy) or deep (typically related to hypertension) but may also be related to rupture of an aneurysm, arteriovenous malformations or other aetiologies, including bleeding diathesis.

Patients require long-term follow-up to monitor adherence with therapy. Typically, this is undertaken in the community, but home-based point-of-care devices and wearables may improve follow-up data collection. Most patients benefit from investigations and preventative interventions after a stroke or TIA, and advanced age is not a contraindication. However, patients with significant frailty, dementia or dependency might be spared some prevention strategies by taking into account their wishes and those of their families. Structured follow-up can not only improve an individualised approach but is cost-effective.^120^

#### State of current services

Provision of secondary prevention services for stroke varies widely across Europe, and guidelines for cardiovascular therapies are inconsistently implemented.^91^ This indicates an urgent need for more accurate monitoring and reporting of secondary prevention across Europe, benchmarked against current KPIs.

Even now, more than 60% of people with a stroke have hypertension, but fewer than 50% of these have adequate blood-pressure control despite high rates of treatment initiation. Only one-third of people after stroke are estimated to have their blood pressure and cholesterol managed to recommended targets, and long-term adherence with preventative strategies is low^92^^,^^114^ despite an increasing number of drugs to help control blood pressure and cholesterol. Further issues concerning access to treatments include speed of access to carotid endarterectomy or stenting, closure of patent foramen ovale or atrial septal defect, left atrial appendage occlusion and long-term cardiac monitoring to detect AF.

Preventative treatments are likely to become increasingly complex and directed to more specific groups of patients. Research into models of delivery of secondary prevention that will lead to Europe-wide standards for secondary prevention comparable to those for acute stroke treatments is needed.

Initiation and adherence with secondary prevention (pharmacological and non-pharmacological) are monitored by quality and outcome control programmes in only a few countries.

Basic secondary prevention is defined in the SST as at least 90% of patients with an indication having access to antithrombotics, antihypertensives, statins and lifestyle advice. Data were based on an estimate in almost all countries, and many countries did not feel confident enough to provide an estimate. Only 8 SAP-E countries reported in the 2022 SST data that access to 3 of the 4 interventions was provided to more than 90% of all stroke patients based on registry data. The situation was best regarding antithrombotics, whereas lifestyle advice seems to be given insufficient attention.

#### Research and development: top 5 priorities

1. Can access and adherence to secondary prevention be improved? (Specific attention to new technologies and approaches, as well as poorly represented underserved groups and long-term follow-up)
2. Can secondary prevention be personalised (eg, through biomarkers and genetic data)?
3. Can we identify specific interventions and approaches that reduce the progression of SVD and its clinical outcomes, including stroke and cognitive decline?
4. Have improvements in best medical therapy changed the threshold for carotid intervention?
5. What is the optimal treatment strategy in patients with AF and a significant risk factor for haemorrhagic stroke (eg, previous haemorrhage or cerebral amyloid angiopathy)?

#### Targets for 2030: top 5 priorities

1. Including secondary prevention in national stroke plans, with follow-up in primary/community care, and ensuring/stimulating translation into local protocols and guidelines.
2. Ensuring that initiation of basic secondary prevention is monitored in quality and outcome assessment programmes.
3. Ensuring that at least 90% of patients have access to basic secondary prevention, including antithrombotics, antihypertensives and statins, as well as lifestyle advice, and that this is monitored (KPI 10).
4. Ensure that at least 90% of the stroke population is seen at a 3–6-month post-stroke follow-up visit (KPI 12a); this can be done by the discharging stroke unit or the general practitioner.
5. Implementing a post-stroke checklist to follow up on secondary prevention, as well as other factors of life after stroke (KPI 12b).

### Rehabilitation

Stroke is the leading cause of new severe disability in adults, affecting daily activities and quality of life. The WHO defines rehabilitation as “a set of interventions designed to optimise functioning and reduce disability in individuals with health conditions in interaction with their environment.”^115^ Rehabilitation aims to enable individuals to live independently and participate in education, work and community life.

Moreover, patients and carers must be involved in decision-making processes and need relevant and understandable information about stroke, rehabilitation, planned discharge and follow-up.^116^

#### State of the art

##### Early stroke unit rehabilitation

Acute stroke care, skilled nursing and specialist rehabilitation in stroke units reduce mortality and disability independent of stroke type.^53^^,^^70^ Rehabilitation in a stroke unit involves occupational, physical and speech therapy, as well as support from psychologists, social workers, dieticians, orthoptists and orthotics, with a multidisciplinary approach including family.^70^

Rehabilitation should always be available in organised stroke units^117^ and remains a cornerstone of stroke unit care. Early mobilisation prevents bed-rest deconditioning but should be tailored to individual needs. Patients should receive rehabilitation therapies (most frequently physiotherapy, occupational therapy, speech language therapy and cognitive therapy) of appropriate intensity and duration, individually designed to meet their needs for optimal recovery and tolerance levels.^118^ Motor rehabilitation should be structured and tailored to provide as much scheduled therapy as tolerated. The training should be meaningful, engaging, progressively adaptive, intensive, task-specific and goal-oriented to improve transfer skills and mobility,^119^ and an appropriate time for restitution should be allowed.

Occupational therapy improves performance in activities of daily living (ADL) and functional mobility through evidence-based strategies such as task-oriented training, self-management strategies, mirror therapy and mental imagery.^121^ Early speech language assessment and intervention for post-stroke aphasia and dysarthria—frequently and with high doses of training—are crucial to maximise language recovery.^122^ In patients with dysphagia, foods and drinks with modified consistency, exercises and optimised positioning for eating and drinking are recommended to improve swallowing function.^108^ Early cognitive screening with further assessment and cognitive training is recommended in patients with cognitive deficits. Patients and their families and caregivers should have early and active involvement in the rehabilitation process, and the training should always be meaningful, engaging and goal-oriented.^118^

##### Rehabilitation after stroke unit discharge

Depending on a person’s needs and mobility, different modes of rehabilitation should be available, as domiciliary, day-case hospital care and home-based care have been shown to improve independence in personal activities of daily living. A personalised transition and rehabilitation plan on discharge is needed to ensure continuity, describe rehabilitation needs and set targets.^123^

Early supported discharge (ESD) offers an evidence-based alternative to continued inpatient treatment, especially in patients with mild-to-moderate stroke.^124^ Indeed, ESD applies to patients with mild-to-moderate neurological deficits and is defined by the rehabilitation being provided by or co-coordinated by a multidisciplinary team. Extra support after ESD showed increased satisfaction with services and seems to reduce resource utilisation (and save costs).^120^ While the optimal duration of rehabilitation varies due to stroke heterogeneity, evidence supports continued rehabilitation for at least 1 year. Continued ADL training at home has shown benefits for up to 1 year after stroke.^125^ Long-term follow-up on functional status and rehabilitation needs is required to identify such needs. Follow-up should take place at least 3–6 months and using a post-stroke checklist to ensure quality by standardisation.^126^^,^^127^

#### State of current services

The WHO made a call for action, “Rehabilitation 2030,”^115^ to address the significant rehabilitation needs across the world and the substantial lack of attention towards these needs shown by many governments. There is considerable variability in access to rehabilitation between and within European countries, likely reflecting differences in the organisation of stroke services, strategic approaches and available resources. Access to organised stroke unit care is still limited in many countries. In 2022, only 11 countries provided access to stroke unit care for at least 75% of hospitalised stroke patients, and access is not monitored or prompt in most countries. No positive developments have been observed since 2021.

The target is access to stroke unit care as the first level of care in at least 90% of patients (KPI 6a). The extensive rehabilitation documented in trials is often absent: In 2022, only 13 SAP-E countries reported that early rehabilitation was provided in approximately 90% of stroke units, with no increase in numbers since 2021 (KPI 9a). ESD is available in at least 90% of stroke units in 3 countries, with no changes from 2020 or 2021 (KPI 9b).

The number and capacity of stroke units need to be increased to ensure that all patients have equitable access to early stroke unit rehabilitation. There is also a shortage of rehabilitation and nursing staff with expertise in stroke and an understanding of rehabilitation.

It is positive that provision of a transition and rehabilitation plan on discharge is increasing: In the 2022 SST data, 17 countries—compared to 14 in 2021—provided rehabilitation to at least 60% of patients (KPI 11). The lack of a transition and rehabilitation plan leads to delays in continuing rehabilitation in the community, limits access to post-stroke support and causes uncertainty for patients and carers about the immediate future after discharge. In 15 countries, stroke services within hospitals, communities and other settings undergo quality auditing continuously or at regular intervals (KPI 5), whereas the quality and outcome control in most countries only covers the hospital sector.

National stroke plans (KPI 1) are needed to define continuity of care and the level of care to be provided and to ensure quality and outcome control in all sectors.

#### Research and development: top 5 priorities

1. Developing evidence-based rehabilitation programmes based on timing, dosing, level, long-term duration and type of intervention.
2. Developing efficient management programmes for fatigue, anxiety and cognitive impairments after stroke.
3. Designing clinical trials, defining how to reach maximal neurological potential in each stroke patient.
4. Documenting the potential benefit of maintenance training.
5. Developing a post-stroke rehabilitation guideline defining best-practice rehabilitation.

#### Targets for 2030: top 5 priorities

1. Providing early stroke unit rehabilitation in at least 90% of stroke units (KPI 9a).
2. Providing ESD in at least 60% of stroke units (KPI 9b) (from the stroke unit or from a community service).
3. Providing a documented individual plan for community rehabilitation and self-management support for all stroke patients with residual difficulties on discharge from hospital to at least 60% of patients (KPI 11).
4. Ensuring that all stroke patients and carers have a review of their rehabilitation and other needs at 3–6 months after stroke and annually thereafter (KPI 12a and KPI 12b).
5. Involving and supporting stroke survivors and their carers during decision-making to ensure that they make informed decisions about their rehabilitation goals.

### Life after stroke

Life after stroke is about helping individuals navigate, adjust to and manage the long-term effects and outcomes of stroke. Historically seen as part of rehabilitation, this domain now stands as a distinct entity, while still acknowledging that these 2 domains are often closely intertwined and complementary. Life after stroke encompasses a wide range of issues and covers children through to very old adults—all with different needs (see Supplementary File File 1). The effects of stroke are huge; key illustrative facts are shown in Table 5.

**Table 5:** Key illustrative facts highlighting the longterm impact of stroke on survivors and their families. The table summarises the prevalence of unmet needs, physical, cognitive and psychological consequences, effects on daily functioning, employment and sexuality, and the burden experienced by caregivers, underscoring the broad scope of the life after stroke domain across all ages.

Each survivor experiences an average of between 2 and 5 unmet needs.^128^ More than 6 in 10 survivors rely on support to help them with daily activities such as getting dressed, making meals or going to the shops.^129^ Post-stroke fatigue affects about 50% of survivors at some point after stroke.^130^ The prevalence of depression is about 30% up to 15 years after stroke.^131^ Risk of suicide attempt and death by suicide in survivors is about twice that of the general population.^132^ At any time after stroke, 1 in 5 survivors will also be living with dementia.^133^ One in 4 survivors is of working age; 1 in 3 will have to give up their job.^129^ More than half of survivors report sexual dysfunction.^134^ Caregivers, and other family member of survivors, have a high risk of developing a mental health condition.^135^

The focus is not merely on helping survivors cope with the life they are left with after a stroke but also on empowering them to live their best possible lives. This involves a holistic approach, encompassing tailored rehabilitation, psychological support and social reintegration. By fostering resilience, independence and quality of life, the aim is to transform surviving into thriving.

#### State of the art

##### Context

“Life after stroke” is an emerging term, and authors are only beginning to use this when classifying papers. Although this is improving, it makes identification of relevant data and evidence problematic in the short term. It is therefore still more difficult to define state of the art in this area, and this section covers specific areas and interventions of interest; good practice examples will be prospectively presented at www.strokeactionplan.org.

##### Transition

Discharge represents a challenging transition. Referrals to services vary widely, and there are no standardised guidelines, although interventions focused on discharge and transitional care could likely improve outcomes and reduce readmissions.^136^

Care plans should be completed in a timely manner before discharge, and patients should be provided with personalised transition and rehabilitation plans on discharge. Where patients have ongoing rehabilitation goals, they should have access to relevant rehabilitation services. Access to re-evaluation and rehabilitation is needed if rehabilitation status changes. Advanced care planning should be in place and reviewed periodically.^137^

##### Function

Key issues include:

- Secondary prevention (covered in Domain 4: “Secondary  Prevention and Follow-up”).
- Post-stroke pain has different causes, including central pain, spasticity and shoulder pain; its treatment requires an individual approach to management.^138^
- Emerging evidence on maintaining function suggests maintenance training and physical fitness programmes can reduce functional decline and offer potential for improvement^139^; however, larger studies are needed to identify the best approaches.
- In post-stroke fatigue**,** underpinning evidence for clinical approaches is lacking,^140^ although several areas show promise. There is now an agreed definition to guide future research.^141^
- Botulinum toxin can be used in rehabilitation of upper limb spasticity,^142^ and other interventions, including extracorporeal shockwave therapy, may provide relief.^143^
- In mental health (including low mood, anxiety and emotionalism through to severe depression), there is a lack of robust evidence for management aside from medication; however, SSOs report that this is a key area for contact.
- Cognitive problems can persist and even worsen years after stroke. Better identification and management are needed.^128^
- Research into aphasia is increasing, with emerging results around communication partner training programmes,^129^ self-management^130^ and specific topics such as managing depression in aphasia.^131^
- Although there is recognition of the effects of providing informal care for survivors, a robust evidence base to address this has proved elusive.^132^ A recent study tested an intervention to support carers, but this did not show any clinical benefits and was unlikely to be cost-effective.^133^ It is therefore still unclear how to effectively support the carers of stroke survivors.
- Follow-up using a structured screening approach, ideally using validated post-stroke checklists, is recommended.^134^

##### Participation (in social, work and leisure time activities)

There is societal evidence around the implications of social isolation,^135^ and social isolation is specifically associated with a higher mortality after stroke.^144^ Participation in social, work and leisure time activities results in wider benefits when activities are seen as meaningful and when there is peer and other support.^145^^,^^146^ There is a moderate association between physical activity and participation levels within the first 6 months following a stroke, with evidence suggesting that this correlation extends beyond 6 months.^147^ However, there are aspects where firm conclusions cannot yet be drawn, such as vocational rehabilitation, although related research shows that fatigue and cognitive deficits are important considerations in planning return to work.^148^

##### Relationships

Anecdotally, SSOs and stroke survivors report that relationships are a problem for many stroke survivors. Sexuality remains a particularly neglected area,^149^ although feasibility research found that peer-supported digital self-management showed potential to progress to a definitive trial.^150^ Education and counselling should be provided.^151^

##### Involvement of people with lived experience

In the spirit of “nothing about us, without us,” stroke survivors, relatives and carers should be involved in all life-after-stroke decisions. They should also receive adequate support, resources and education tailored to their unique needs.^152^ While measuring the success of such involvement may be challenging, it represents an essential cornerstone of good practice, fostering collaboration, empowerment and improved quality-of-life outcomes.

##### Supported self-management

Self-management support is not widely available, although there is growing interest that it may improve quality of life and self-efficacy.^153^ Group self-management interventions have been shown to increase knowledge, collaboration, goal setting and problem solving. Peer support-facilitated interventions promote sharing experiences, vicarious learning and increased motivation.^154^^,^^155^ There has also been much interest in the results from an RCT of a low-cost, person-centred, self-directed intervention—the Take Charge Programme—which improved quality of life and independence.^156^

#### State of current services

Provision of long-term life-after-stroke support varies widely between, and within, European countries. In many countries, data related to long-term outcomes have not been collected comprehensively, systematically or rigorously, and there are still countries where no adequate information or data are available. There are therefore relatively few robust datasets; some of these are presented in Supplementary File File 2.

Most stroke survivors experience unmet needs. Mitigating these needs is hampered not only by lack of resources but also by the weak evidence base and inconsistencies in reporting**.** Further work is required to understand how promising interventions translate and are implemented into practice across countries and stroke pathways. According to the 2022 SST data, only 11 countries provided a life-after-stroke programme in 2022. In the short term, formal services, SSOs and voluntary groups have great potential to evaluate their contributions to life after stroke and share best practices.

##### Formal services

Community services and post-hospitalisation care need to be improved and organised in the same way that improvements have been made in acute hospital care. A large systematic review concluded that “comprehensive and pragmatic programs operated by the multidisciplinary stroke team hold promise to reduce the long-term health burden of stroke.”^157^ The role of a key worker, navigator or coordinator has shown success in terms of improving satisfaction, and while evidence specific to stroke is limited, there is some evidence of patient navigation supporting better coordinated care.^158^

##### Third-sector organisations

Survivors often seek support outside of formal health and care systems^134^ to access advice lines, specific support or peer support provided by, for example, SSOs. The network of SSOs globally is growing, with opportunities for shared learning, implementation research and advocacy. Despite the lack of a strong evidence base, there are anecdotal reports of benefits of life-after-stroke support in its widest context. Few opportunities for good practice to be shared exist. The newly formed Stroke Alliance for Europe’s European Life after Stroke Forum^159^ may be 1 solution, but much more needs to be done, as we still do not have models of what best care looks like.

##### Areas of immediate and significant challenge

###### Long-term follow-up

Despite emerging evidence on the value of conducting regular reviews to identify longer-term needs and trigger referrals, implementation of regular review in practice remains inconsistent. For example, although the UK’s clinical guidelines for stroke^152^ strongly endorse 6-month reviews, just 37% of stroke survivors in England and Wales in 2022-2023, had a review at 6 months.^160^

The lack of data collected on outcomes is compelling and has significant implications for individuals and for the strategic planning of support for stroke survivors. Recent research has reaffirmed the importance of addressing longer-term unmet needs of people with stroke, but effective evidence-based models of care are not yet available.^161^

###### Transition and care

Many stroke survivors continue to feel unsupported (“abandoned”) after leaving hospital care,^162^ and transitioning between hospital and community and within community services is often particularly challenging. Survivors consistently report needing help to optimise recovery and secondary prevention.^163^ According to the 2022 SST data, 17 countries provided a transition and rehabilitation plan at discharge to at least 60% of stroke survivors in 2022; however, only 4 countries support this with registry data. This is an improvement from 2021, where a plan was provided in 14 countries.

Of note, severe stroke may involve end-of-life support. One study found that most people with severe stroke, even those who die in hospital, do not receive palliative care consultations.^164^ However, again, there is limited evidence and guidance regarding best practice.^165^

###### Participation

No robust data are available specifically on the status of participation in social, work and leisure time activities after stroke. However, based on reports of unmet need,^120^^,^^166–168^ significant challenges must be assumed. Recent research has found a mismatch between the needs reported by stroke survivors and evidence available on how to address these needs.^161^ The timing and content of interventions to support longer-term participation and recovery require further focus.

###### Relationships

In some countries, written information and workshops for stroke survivors and families have been developed by SSOs.^169^^,^^170^ However, overall, robust evaluation of the uptake or success of these is lacking.

###### Involvement

Involvement of stroke survivors and their families in care plans is generally recommended in stroke pathways. However, there are no data on the execution or quality of this involvement.

###### Carers

We know that the care burden is heavy. For example, in research conducted in Sweden, outcomes were documented for 5053 informal stroke caregivers at 3- and 5-year follow-up.^171^ Among those supporting completely dependent survivors, less than half (49%) received support and 24% expressed an unmet need for support. However, despite such statistics, we still have relatively little to offer in terms of evidence-based interventions.

###### Awareness

Campaigning to raise awareness may also improve traction with policymakers. The UK Stroke Association launched a recent campaign on “Thriving not surviving” to increase public knowledge of the struggles stroke survivors face.^172^ Such initiatives are important to facilitate recognition by society of the worth and value of those with disabilities.

#### Research and development: top 5 priorities

1. What are the experiences and needs of stroke survivors at different times during their lifespan, considering different cohorts of stroke survivors and challenges of those with multiple morbidities—and their carers—to inform the design of optimal care pathways?
2. What would a model of best care and long-term support look like? This should include the opportunity for reviews and specific roles to provide holistic, coordinated support.
3. How can data on life after stroke best be collected within stroke registries to improve understanding of the long-term outcomes of stroke and service planning, and what data should this comprise?
4. What products and services (digital and physical) would support self-management, community integration, education and healthcare?
5. How can high-quality information and training to help non-specialist staff, especially social care staff, be targeted? It is envisaged that this will involve research around staffing levels, core competencies and the involvement of non-governmental and non-profit-making bodies such as charities and voluntary groups.

#### Targets for 2030: top 5 priorities

1. Providing comprehensive stroke follow-up that addresses all aspects relevant for life after stroke (KPI 12a).
2. Using a recognised post-stroke checklist and functional assessment to capture all stroke-related health problems. People should be referred to as appropriate (KPI 12b).
3. Providing equitable support, established through national stroke care plans and in conjunction with SSOs, to stroke survivors, regardless of their place of residence and socioeconomic status. Minimum standards should be agreed for what every stroke survivor should receive regardless of where they live (KPI 1 and KPI 2).
4. Ensuring appointment of government-level individuals or teams responsible for inclusion of life after stroke in national stroke plans, with supporting national databases in place for quality improvement.
5. Exploring implementation of supported self-management information and assistance systems needs as a priority area.

### Evaluation of outcomes and quality improvement

Quality of stroke care across Europe exhibits significant variations between and within countries. These disparities can be attributed to uneven access to medical resources and how healthcare services are organised. The SAP-E platform supports the SST—a tool to monitor and benchmark countries' performances through a given set of stroke metrics and indicators.^12^ The SST is meant to complement national and international registries, which are necessary to support organisations in measuring indicators in clinical and organisational practice.^173^

#### State of the art

##### Guidelines

The ESO guidelines^174^ are generated according to the Grading of Recommendations Assessment, Development and Evaluation system according to a defined Standard Operating Protocol,^175^ at times in collaboration with other scientific societies. The ESO guidelines serve as an exemplary international framework; however, it is essential to customise these guidelines to align with the specific needs and structures of various national health systems and with existing regulations and standards to guarantee superior quality and compliance in service delivery. A recent review of updated stroke guidelines at the global level and in multiple language–collated and –matched recommendations to the level of service available,^176^ also advising strategies to drive forward service development. In recent years, the living guideline development approach^177^ has emerged to integrate new evidence into recommendations in real time.

##### Stroke service certification

Certification of stroke services provides an objective assessment of stroke infrastructures, creates a cohesive team and recognises professionals’ contributions^178^ in certified institutions. Stroke centre certification programmes have been associated with lower mortality, improved functional outcomes and improved guideline concordance.^179^ Certification is provided by several different agencies/bodies: independent organisations such as ESO and national/international organisations. ESO has established a Stroke Unit and Stroke Center Certification programme^107^ that supports healthcare organisations to provide a consistent approach to care, reducing the risk of errors. However, guidelines and quality metrics for stroke care certification vary,^180^ and this variability underscores the importance of establishing and adhering to high standards to ensure good patient outcomes.

##### Measuring quality

Quality should be measured both at the point of care and at the health-system level. Clear, consistent standards, shared indicators and evidence-led assessment of the quality of stroke services are essential if quality improvement is to be achieved. In addition, results of quality monitoring, with appropriate interpretation, should be made available to patients and the public to provide assurance that high-quality care is being delivered and to act as a driver of improvement within the system. If valid and reliable comparisons are to be made between and within stroke services nationally and internationally, it is essential that definitions and terminology are agreed and standardised across countries.

One of the reasons for apparent differences in incidence and outcomes after stroke is that different countries collect and report data with different accuracy. This variability has several causes: (1) variation and miscoding of International Classification of Diseases (ICDs) codes, because countries are using different versions of the ICD (ICD-11 launched in 2022 is in use in 35 countries globally) and because of miscoding due to inadequate training of healthcare professionals, (2) standards for collecting comparable data differ among countries,^181^ (3) the operational conceptual framework of stroke measures may vary,^182^ (4) outcome measures in place are scarce and (5) data quality and completeness vary.

In recent years, international initiatives have promoted the implementation of stroke registers, eg, the Registry of Stroke Care Quality (RES-Q)^183^ and *Safe Implementation of Treatments in Stroke*.^184^ International and national registries are mainly institution-based and traditionally focus on the management of the acute phase of stroke, access to reperfusion therapies, hospitalisation in stroke units, diagnostic work-up and secondary prevention. Recently, post-discharge and follow-up data are obtained through digital medicine solutions that make modified Rankin Score and other functional and quality-of-life metrics available.

In some cases, as in Slovakija,^185^ data are imported from the national/governmental registry, allowing a population-based approach of registry data collection. This approach is preferred, where possible, to ensure completeness.

##### Supporting improvement through clinical audit

Audits are necessary but must be sustainable. Audit models should be selected based on sustainability, as well as available resources (continuous, intermittent or snapshots). A valuable recent innovation in audit is the involvement of patient interest representatives in supervision and design of audits.

##### Evaluation of stroke outcome

Traditionally, important clinical outcomes after stroke have included survival, stroke recurrence and the need for long-term aftercare, as well as a diverse range of measures that quantify the direct and indirect impact of stroke on patient functioning. Supplementary File File 3 presents widely used evaluation tools, including the Patient-Reported Outcomes Measurement Information System, which is used along the continuum of stroke care.

#### State of current services

**Figure 4:**
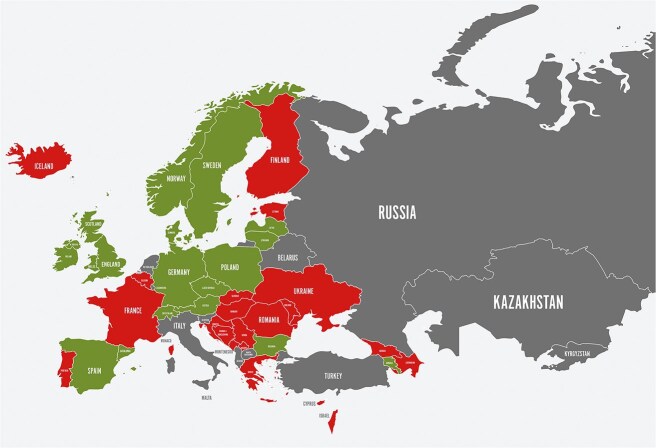
Countries are categorized by color as follows: green—Norway, Sweden, Denmark, Germany, Poland, Spain, Italy, United Kingdom (England and Scotland), Ireland, Estonia, Latvia, Lithuania, Bulgaria, and Armenia; red—Iceland, Finland, France, Ukraine, Romania, Greece, Cyprus, Israel, Georgia, and Azerbaijan; gray—Russia, Belarus, Turkey, Kazakhstan, Kyrgyzstan, Serbia, Bosnia and Herzegovina, North Macedonia, Albania, Kosovo, Montenegro, Moldova, Malta, and Monaco.

National quality guarantees for health services—specifying the level of competence and user experience that patients can expect along the continuum of stroke care—vary among European countries. According to the 2022 SST data, 17 European countries have national stroke plans defining pathways, care and support after stroke, including pre-hospital phase, hospital stay, discharge, transition and follow-up (KPI 1). National or regional services for quality improvement and assessment (KPI 4) are still underrepresented, with 30 SAP-E countries still lacking accountability in stroke care (Figure 4). Few European countries report stroke units and stroke services—independent of the organisational sector—undergoing quality auditing continuously or with regular time intervals (KPI 5). However, regular quality auditing of stroke care seems to happen more frequently in hospital facilities and in the acute phase of care, while community stroke services providing post-acute, rehabilitation and long-term care, including palliative care, are less likely to be audited. Many healthcare systems have a stroke quality improvement programme in place, with national registries,^186^ national guidelines (often available in plain language), audits and certification as quality improvement tools. A recent helpful initiative is the WHO Office on Quality of Care and Patient Safety, which was established in 2021 and aims to improve the quality of care and patient safety in Europe.

National guidelines for stroke management have been produced in many countries and, in most cases, are aligned with the ESO guidelines covering all areas of stroke care—from primary prevention to rehabilitation and long-term consequences of stroke and including acute stroke, prevention and management of complications and secondary prevention. Full-text guidelines can be downloaded from the guidelines repository, including videos summarising the evidence.^174^ Furthermore, the SAP-E website includes the Essentials of Stroke Care, a document written as a tool for SAP-E by a working group appointed by the ESO Guideline Board. This document is meant to provide an overview of evidence-based interventions covering the entire chain of stroke care.^187^

Few international comparisons of care are based on high-quality data. The Global Burden of Disease (GBD) study shows large variations in case-fatality rates within Europe, which range from 3% in Denmark to 18% in Latvia.^1^ However, the quality of GBD data varies significantly between European regions, weakening the validity of this dataset. The SST^31^ aims to fill this gap by creating a European overview of performance against SAP-E KPIs, stroke care organisation, pathways, stroke incidence and early mortality.

Clinical audit has become an essential part of the quality improvement cycle. There has been a gradual evolution, with increasing use of stroke registers—which are more common than national audits—for audit and quality improvement purposes and selection of data items that reflect areas where standards or guidelines for care exist. The 2022 SST data show that national quality registers are available in some countries and regions, including Austria, Catalonia, Czechia, Denmark, Finland, Germany, Ireland, Israel, the Netherlands, Norway, Slovakia, Sweden and the United Kingdom (SST 2022 data), but are of varying quality regarding data completeness and correctness. The RES-Q, a quality register initially designed to support Eastern European countries, is now operating at the global level and has become a useful tool to capture quality at the point of care and, most recently, at follow-up in RES-Q version 2.0. At a national level, only Sweden, Denmark and the United Kingdom provide data on transition and functional outcomes. Very few countries make quality data available to the general public. Quality improvement is structured regionally rather than nationally in many countries, including Spain, Finland, Portugal and Italy, which can result in significant within-country variations in care quality. Quality indicators for stroke have been published and are regularly updated in Italy,^188^ Sweden,^189^ Norway, Denmark,^190^ the Republic of Ireland, the United Kingdom, Germany, Portugal, France and Turkey. A consensus paper on core standards for measuring quality has been published.^191^

#### Research and development: top 5 priorities

1. What definitions should be used across Europe for recording and reporting of data on stroke and TIA?
2. How can data on the quality of care be used to compare process and outcomes of care, taking into account variations in case-mix, and what is the minimum dataset that is needed?
3. What sustainable systems are needed to allow international comparisons of the clinical and cost-effectiveness of care and reporting of within-country variations and variations by other factors such as geographical region (urban vs rural) and over time?
4. Which strategies support the effective use of clinical guidelines and clinical quality registry data to inform health/stroke service delivery?
5. How can new technologies be used to (1) extract audit or register data automatically from electronic patient records to reduce burden on stroke services and increase consistency in data collection and (2) to conduct simultaneous data evaluation in multiple national and regional registers without the need to transfer large datasets and without data protection issues?

#### Targets for 2030: top 5 priorities

1. Defining a common European framework of reference for stroke care quality, including:
  - Strengthening the development of updated European guidelines for management of acute stroke care, longer-term rehabilitation and prevention; where appropriate and sustainable, the living guideline model could be adopted.
  - Expanding and implementing the SST as the tool to enable accurate international comparisons of care at the health system level in the hospital and in the community (including structure, process, outcome measures and patient experience).
  - Assigning a named individual who is responsible for stroke quality improvement in each country or region.
2. Defining a common European framework of patient metrics and variables reflecting quality indicators, including:
  - a minimal dataset that should be provided as a part of patient documentation
  - a data dictionary that defines quality indicators
  - a list of recommendations to ensure interoperability between different national and international registries.
3. Establishing national- and regional-level systems for assessing and accrediting stroke clinical services, providing peer support for quality improvement and making audit data available to the public (KPI 4).
4. Regular certification or equivalent auditing processes for quality improvement of all stroke units and other stroke services (KPI 5).
5. Collecting patient-reported and longer-term outcomes (eg, 6 months and 1 year), covering hospital and community care, considering digital health solutions for this purpose (eg, web apps).

### Translational stroke research

#### State of the art

There have been substantial advances in understanding of the pathophysiology of stroke and chronic cerebrovascular diseases,^192^ but translating this knowledge into successful treatments has been unsatisfying. Technological developments now offer new opportunities to decipher pathophysiological processes underlying cerebrovascular diseases, identifying novel targets for drug therapies and drug repurposing, particularly for prevention and maintaining vascular health and stroke recovery. In light of this, interest is increasing in personalised medicine approaches using genomics, imaging and other biomarkers for stratification of stroke patients. However, novel opportunities run the risk of failing in translation, unless accompanied by changes in the way research is performed.

Bridging the “translational gap” between basic and clinical stroke research is critical for the development of effective treatments. Key requirements include improved networking between basic scientists and clinicians, better experimental designs, development of more relevant experimental models that mirror the complexity of human diseases and applying similar rigour in animal studies to that in clinical trials, such as multicentre evaluation and double blinding.^193–195^

Supplementary File File 4 highlights major topics where recent advances offer new opportunities for translational research, focusing on mechanistic studies and development of novel therapies in relation to stroke prevention, acute stroke, secondary injury, recovery and rehabilitation and life after stroke.

#### Current state of research

A number of open questions remain in the current pipeline of translational research. To overcome the obstacles to successful translation requires several key strategic measures; Table 6 summarises action points aimed at changing conventional practice by designing novel strategies to tackle translational bench-to-bedside research.

**Table 6:** Strategic action points to improve translational stroke research and facilitate successful bench-to-bedside translation.

**Strategic step**	**Action points**
Exploratory vs confirmatory studies	High-quality basic research with: focus on hypothesis testing state-of-the-art, rigorous methodology transparency in methods and results data availability for sharing (including deposition of protocols/data in public repositories)
Preclinical confirmatory studies as an intermediate translational step towards clinical trials	Discovery and confirmation in separate studies Preclinical confirmation before undertaking clinical studies Pre-registration of animal studies Pre-planned study designs and analyses Publication of all results (including negative results)
Improve experimental modelling	Use models resembling human condition Reduce bias Increase power Include comorbidities Apply more variability (eg, genetics, different habitats, ageing, sex)
Change to a larger “team” concept	Establishing “team science” in stroke research Use multiple sites in preclinical trials corresponding to multicentre studies
Improve efficacy of early-stage clinical trials	Regulations should be more proportionate to the risks of the trial Carefully stratify patients for clinical trial inclusion, with the perspective of developing future personalised treatment
Triangulation of evidence to select targets for clinical testing	Seek evidence from: multiple methods (eg, studies in animals with gain- or loss-of-function mutations/pharmacological targeting, epidemiology, Mendelian randomisation, human tissues) multiple data sources (eg, different laboratories, populations and environments) multiple investigators

#### Exploratory vs confirmatory studies

Preclinical research has traditionally lacked confirmatory studies to test the efficacy of treatments. Instead, exploratory studies to identify and investigate new molecular or cellular pathways and mechanisms have been combined with therapeutic experiments that are hampered by insufficient statistical power and poor design, resulting in low reproducibility.

#### Preclinical, confirmatory studies as an intermediate translational step

Clinical trials based on preclinical target identification and drug development have typically relied on small-scale, single-centre studies that are statistically underpowered. As a result, current translational efforts represent a huge leap from small exploratory studies to large confirmatory trials in a highly variable human disease. This gap requires an intermediate step to improve the reliability of translational research and solve the problem of lack of replication.

#### Improve experimental modelling

An important reason for the failure of translation to date is the lack of internal, external and construct validity of current experimental modelling. Experiments are mostly conducted on young, male, genetically identical rodents housed in artificial, pathogen-free conditions and performed under anaesthesia. As a result, they may not accurately reflect the variable conditions encountered in clinical medicine.

#### Adopt a “team science” approach

Large-scale collaborations with a “team science” approach are needed to guide further development in translational research. Clinicians should partner with basic researchers by specifying research needs and contributing to the design of preclinical studies from a clinical perspective. Initiatives for large-scale preclinical multicentric trials using, as far as possible, protocols that are generally accepted for clinical trials are underway, but the utility of this tool has yet to be proven.

#### Improve efficacy of early-stage clinical trials

Early-stage clinical trials represent an intermediate step between pre-clinical drug development and large-scale clinical trials. As such, the designs should keep up with the pace of preclinical target identification, be sufficiently sensitive to test novel approaches in a stratified, optimal target population, and, when possible, seek a genetic rationale for drug effects.

#### Triangulation of evidence to select targets for clinical testing

Candidate drug targets that rely on multiple methods (eg, studies in animals with gain- or loss- of-function mutations; pharmacological targeting in experimental animals, epidemiology including population-based studies, Mendelian randomisation and human tissues), multiple data sources (eg, from different laboratories, populations and environments) and multiple investigators have a greater chance of success in clinical trials. Investigators should consider the principle of triangulation when selecting targets for exploration in proof-of-concept studies in humans or larger clinical trials.

#### Involvement of various parties

The EU and national funding bodies must commit to investing in stroke research on a scale commensurate with the magnitude and prevalence of the health problem. Strategies to validate results from exploratory research require a collective effort that goes beyond the capacity of individual projects or small, sporadic collaborations. Strong independent institutional support is needed to make the transition from traditional designs to a novel concept of organised research structures and data validation in order to facilitate reliable translation of pre-clinical findings to clinical practice. The pharmaceutical and medtech industries should be involved in this process: This could be achieved by facilitating exchange between academic and pharmaceutical research in the transition from exploratory to confirmatory preclinical studies. Finally, researchers need to disseminate their findings to bring stroke research closer to patient advocacy groups and the general population.

#### Targets for 2030: top 4 priorities

1. Creating an organisational framework by implementing confirmatory pre-clinical research through “team science” and by providing novel tools for advanced trial designs to increase validity.
2. Developing and implementing guidelines for preclinical stroke studies on new treatments to maximise the success of clinical translation.
3. Focusing experimental stroke research on identifying new treatable targets with high translational potential that will lead to successful clinical trials by 2030.
4. Identifying novel therapeutic targets for subtypes of stroke with no specific mechanistic treatment available to date, especially cerebral SVD and ICH.

## Discussion

This mid-term review of the SAP-E has resulted in significant updates in the sections on state of the art and state of current services and research, as well as development of new KPIs.

### Primary prevention

The main finding for primary prevention is the large unmet potential for primary stroke prevention, especially regarding the increasing burden of hypertension and metabolic risk factors, whereas the impact of tobacco is decreasing. To resolve this unmet potential, interventions must address all age groups and focus on physical activity, diet, alcohol and tobacco, as well as opportunistic screening for hypertension. Cost-effective interventions that have been identified should be put in place, and monitoring systems should be implemented at all levels to ensure progress. This has led to a more ambitious overarching target for stroke reduction of 15% (2018-2030), which is now based on the age- and sex- standardised rate, thereby taking the ageing population of Europe into account.

### Access to organised stroke unit care

The main aspects from the stroke care domain are significant inequity in access to stroke unit care and quality of stroke unit care. Admission to stroke unit care and the timing of this are only monitored in some countries. DRG coding covering admission to organised stroke unit care could mitigate this. Even if patients have access to stroke units, early stroke unit rehabilitation is often not provided or not available in a timely manner, as disciplines such as speech language therapists and occupational therapists are not represented in stroke units. Early rehabilitation includes preventing complications following bedrest deconditioning in severe stroke, as well as initiating long-term rehabilitation.

### Management of acute stroke

Clear protocols and pathways are needed for time-dependent therapies and should include the pre-hospital sector and be based on the regional situation. Despite scientific developments in hyperacute stroke treatments, accessibility of IVT and endovascular treatments such as MT varies considerably, although the situation has improved in recent years when looking at Europe as a whole. However, considerable inequity in treatment rates persists between countries. Developments in acute treatment have been centred around acute ischaemic stroke, with a risk of leaving ICH behind despite scientific developments in this condition. A specific KPI for ICH mortality has been added to monitor acute care in ICH.

### Secondary prevention and follow-up

All major pharmacological interventions in secondary prevention of stroke are now listed as WHO “Best Buys.” However, only a few countries monitor initiation of secondary pharmacological prevention, and lifestyle interventions are not monitored in any country. This requires action, as about 25% of all admitted strokes are recurrent strokes, and the vast majority of patients present with significant uncontrolled risk factors.^196^ It is likely that providing systematic follow-up after stroke, including monitoring of secondary prevention, would support patients and reduce the impact of fragmentation of healthcare systems. National stroke plans should clearly define pathways, responsibilities and monitoring of secondary prevention, including at least 3–6-month follow-up post-stroke with a standardised checklist. Research into better and more personalised secondary prevention strategies is also needed.

### Rehabilitation

Rehabilitation is now an essential intervention—as defined by the WHO—and is supported by strong evidence. However, only a minority of European patients with stroke have access to any or adequate rehabilitation. Rehabilitation is poorly monitored, often because of fragmentation in healthcare systems. We therefore need to build up facilities for stroke rehabilitation and ensure that an individual plan for rehabilitation and self-management support is available on discharge from the stroke unit. To further improve efficacy of rehabilitation, there is a significant need for adequately sized clinical intervention studies to guide cost-effective practice in rehabilitation, especially regarding methods and dosing.

### Life after stroke

Life-after-stroke services and support are essential to enable those affected by stroke to navigate, adjust to and manage the long-term effects of stroke. Only with structured follow-up—addressing survivors’ practical, social, emotional and clinical needs after hospital discharge—can they (and their carers) live the best life possible. However, the funding of such services and research to maximise their impact is routinely under-prioritised.

### Evaluation of outcomes and quality improvement

The quality and outcome assessment domain documents a need for a standard European definition of a valid programme being in place as part of a national stroke plan or otherwise. One crucial aspect is coverage of monitoring, what proportion of patients are admitted to institutions with monitoring programmes (certification and registries). The national stroke plan must ensure the means to follow basic information on patients treated outside the monitored stroke system. When assessing the quality of stroke care in a country or a region, the denominator should be all strokes, independent of the site of admission. The SST visualises apparent gaps in a large proportion of countries. This includes data quality and timeliness of data, as well as important steps not being monitored. One example is admission to stroke unit care or the timing of this.

Furthermore, monitoring is almost exclusively in place in hospital sector institutions, although most rehabilitation is provided outside of the hospital sector and transfer occurs within the first 3–7 days in many countries. Consequently, monitoring of the chain of stroke care is hampered by the fragmentation of healthcare services, especially in case of rehabilitation outside of hospitals and follow-up. Presently, a significant proportion of European countries still lack systems for quality and outcome control in stroke and only few countries have datasets that allow for data-driven governance at a national or regional level. Quality and outcome control are needed to ensure equity and quality in care; improvements in this area will lead to improvements in all other domains and should be anchored in a national stroke plan.

### Translational research

Bridging the “translational gap” between basic and clinical stroke research is critical for the development of effective treatments. Key requirements here include improved networking between laboratory scientists and clinicians, better experimental designs, development of more relevant experimental models that mirror the complexity of human diseases and applying rigour in animal studies similar to that in clinical trials, such as multicentre evaluation and double blinding.

To ensure development of better interventions in stroke, clinical intervention studies must be of high quality following accepted standards (eg, Consolidated *Standards* of Reporting Trials [CONSORT]^197^). Clinical trial networks and international collaboration between clinical trial networks (eg, ESO Trials Alliance^198^) facilitate the conduct of high-quality clinical trials. Trials must be adequately sized to answer a clinically relevant research question, and bias must be controlled. Patient representatives must always be included in the planning and needs defined by patients (eg, James Lind Alliance *Stroke Priority* Setting Partnership^199^) should be prioritised.

### Clinical research

Use of new technologies may contribute to cost-effectiveness but must also comply with digitisation of the target population and General Data Protection Regulation–based restrictions in data transfer. Reliability is a high priority, as well as integration into other existing digital systems. Methods in clinical intervention studies should allow for and focus on easy implementation in different healthcare systems. Further advancement of the stroke field must continue to be developed using high-quality scientific methods and focus on health-economic aspects to bring about societal change but must also be aligned with patient-centred perspectives to ensure that delivery of future healthcare services is in accordance with patients’ needs. Strategic steps for research in stroke are proposed in Table 7. To meet the top 5 prioritised research and development targets in the seven domains of stroke, a significant increase in funding of stroke trials adhering to this outline is needed.

**Table 7:** Strategic research and development priorities for advancing stroke care across the continuum.

Cost-effective approaches to primary prevention, especially in high-risk populations Further developments of acute interventions, especially in ICH Development of complex interventions that are cost-effective in increasing long term adherence to secondary prevention Development of secondary prevention interventions based on stroke aetiology Development of well-defined, cost-effective and efficient interventions in rehabilitation (motor, speech language, cognition) with focus on dosing and implementation Developing complex interventions that are cost-effective in increasing long term quality of life after stroke for patients and carers Improve experimental modelling, networking among scientists and efficacy of early-stage clinical trials

### Conclusion

Stroke remains a significant health issue in Europe, with notable incidence and inequities in access to care. Key interventions along the stroke care pathway are strongly evidence-based and supported by WHO and EU recommendations (eg, “Best Buy” list, Healthier Together and EU Best Practice Portal). Despite improvements, gaps remain across the care pathway but particularly in terms of access to stroke units, rehabilitation, follow-up care and secondary prevention.

## Supplementary Material

aakaf026_Supplemental_Files

## Data Availability

Not applicable.
