# Correction to: Stroke Action Plan for Europe 2018–2030 (SAP-E): mid-term review and update

**DOI:** 10.1093/esj/aakag081

**Published:** 2026-06-30

This is a correction to: Hanne Christensen, Francesca Romana Pezzella, Melinda Berg Roaldsen, Aleš Tomek, Arlene Wilkie, Louisa Christensen, Martin Dichgans, Avril Drummond, Tiina Laatikainen, Carlos A Molina, Katharina S Sunnerhagen, Danilo Toni, Sonia Abilleira, Diana Aguiar de Sousa, Anita Arsovska, Heinrich Audebert, Jelena Bartolovic, Yannick Béjot, Geert Jan Biessels, Juliet Bouverie, Hrvoje Budincevic, Barbara Casolla, Hugues Chabriat, Marina Charalambous, Jesse Dawson, Stephanie Debette, Frank-Erik de Leeuw, Adam Denes, Marina Diomedi, Diederik Dippel, Ulrich Dirnagl, Urs Fischer, Yuriy Flomin, Ana Catarina Fonseca, Birgitte Forchammer, Anne Forster, Giovanni Frisullo, Miquel Galofre, Zuzana Gdovinová, Christoph Gumbinger, Joseph Harbison, Richard Hobbs, Dalius Jatuzis, Hrvoje Jurlina, Mira Katan, Lisa Kidd, Stefan Kiechl, Janika Kõrv, Christina Kruuse, Wilfried Lang, Arthur Liesz, Svetlana Lorenzano, Andreas Luft, Grethe Lunde, Chris Macey, Hugh Stephan Markus, Gillian Mead, Patrik Michel, Serefnur Ozturk, Maurizio Paciaroni, Aleksandra Pavlovic, Carina U Persson, Terence J Quinn, Peter Rothwell, Luca Saba, Paola Santalucia, Gustavo Santo, Claus Simonsen, Thorsten Steiner, Katarzyna Stolarz-Skrzypek, Cristina Tiu, Alexander Tsiskaridze, Georgios Tsivgoulis, Jaakko Tuomilehto, Teresa Ulberg, Paolo Ursillo, Antonella Urso, Mia van Euler, Margus Viigimaa, Denis Vivien, Markus Wagner, Marion Walker, Alastair Webb, Diana Wong Ramos, Mauro Zampolini, Marialuisa Zedde, Gary Ford, Peter Kelly, Robert Mikulik, Bo Norrving, Hariklia Proios, Simona Sacco, Else Sandset, Joanna Wardlaw, Aleksandras Vilionskis, Valeria Caso, on behalf of the SAP-E collaborators, Stroke Action Plan for Europe 2018–2030 (SAP-E): mid-term review and update, *European Stroke Journal*, Volume 11, Issue 1, January 2026, aakaf026, https://doi.org/10.1093/esj/aakaf026

In the originally published version of this manuscript, author Teresa Ullberg’s name was misspelled.

This error has been corrected.

