# Supplementary material for: Stroke Action Plan for Europe 2018–2030 (SAP-E): mid-term review and update
Source: Eur Stroke J. 2026 Jan 19;11(1):aakaf026. doi: 10.1093/esj/aakaf026 (PMC12866651; doi:10.1093/esj/aakaf026)
Supplement: aakaf026_Supplemental_Files [file aakaf026_supplemental_files.zip › Supplementary_file_1_aakaf026.docx]

# Supplementary file 1: Challenges in life after stroke for specific age groups

Although we use the general term life after stroke to apply to everyone who has had a stroke, the specific challenges in specific age groups need to be recognised. The needs of people with stroke are very different, and age is an important consideration.

## Children and young people

There is no Europewide agreed model for long-term support of children or young people who have had a stroke. There is a clear lack of data and, anecdotally, it seems that care in many countries is provided by generic or neurological services with no clear evidence of ongoing holistic support. Where services are available, they can be fragmented, and the transition from child to adult services is often disjointed. As with many paediatric medical diagnoses, there is no clear pathway to access ongoing adult services and support. There is evidence of some good practice – for example, the Danish Department of Health issued guidelines for rehabilitation and support for children and young adults.^1^ The UK stroke clinical guideline also includes a recommendation, which states that a system needs to be in place to ensure a seamless transition for younger people with stroke from paediatric to adult neurological services,^2^ but, although this is agreed, this could be operationalised in practice is not clear.

Since 2016, the Swedish Pediatric Stroke Register (Barnriksstroke)^3^ – which forms part of the national stroke register (Riksstroke) – aims to improve the quality of care for children and young adults who have had a stroke, to minimise the time between diagnosis and treatment, and to guarantee that children's investigation, therapy and rehabilitation are of high and consistent quality across the country. However, overall, good models and strategy are lacking.

## Midlife

In midlife, the life after stroke needs of stroke survivors again differs from those of other ages. There is often a greater need for personalised support and intervention around employment, family caregiving and other relationships. Yet generic stroke support services are often structured around the needs of older adults beyond retirement age. Such services are a poor fit for younger and midlife stroke survivors and can lead to disengagement and frustration, although these services can meet the needs of some. Again, the Danish Department of Health has issued guidance around rehabilitation for stroke and traumatic brain injury and life after stroke.^4^ Although these guidelines have not been fully implemented, this has been a valuable tool for strategic planning and for policymakers and patient-facing organisations. It is therefore thought to have been a worthwhile endeavour. Other countries provide several examples of clinical guidelines and national recommendations.

## Older adults

Most people living with stroke are older adults, but older adults are not a homogenous group, and each person will have different needs following stroke. Increasing life expectancy has not been accompanied by universal healthy ageing, and many stroke survivors are living with frailty or complex comorbidity. A recent review suggested that one in four people presenting with acute stroke have a background of frailty and another two-thirds are at risk of developing frailty.^5^ For this group, an exclusively stroke-focused approach that does not account for their other issues or comorbidities is at best reductionist and at worst could cause harm.^5^ Research and policy need to design and implement structures for life after stroke for frail, older adult stroke survivors.

## References

1. Danish Health Autority. Forløbsprogram for rehabilitering af børn og unge med erhvervet hjerneskade. 2011, p. 90.

2. Party ISW. National Clinical Guideline for Stroke for the UK and Ireland.

3. Riksstroke. The Swedish Stroke Register, <https://www.riksstroke.org/sve/barnriksstroke/>.

4. Danish Department of Health. Recommendations for cross-sectoral programmes for adults with acquired brain injury. 2020.

5. Burton JK, Stewart J, Blair M, et al. Prevalence and implications of frailty in acute stroke: systematic review &amp; meta-analysis. *Age Ageing* 2022; 51. DOI: 10.1093/ageing/afac064.
