# Supplementary material for: Stroke Action Plan for Europe 2018–2030 (SAP-E): mid-term review and update
Source: Eur Stroke J. 2026 Jan 19;11(1):aakaf026. doi: 10.1093/esj/aakaf026 (PMC12866651; doi:10.1093/esj/aakaf026)
Supplement: aakaf026_Supplemental_Files [file aakaf026_supplemental_files.zip › Supplementary_file_2_aakaf026.docx]

# Supplementary file 2: Cohort studies for life after stroke

Ongoing data collection in several countries has informed or has the potential to inform life after stroke agendas.

An excellent example of systematic and long-term data collection is the Swedish Stroke Register,^1^ which was the world’s first nationwide quality register for stroke care and is still in use. Since 2008, the register has covered quality of services and late outcomes after stroke through follow-up, including at one year after stroke. Analysis of these longer term data found that about 16% of stroke survivors deteriorated in function from independence to dependency between three and 12 months^2^ and that one in five had unmet rehabilitation needs at 12 months post-stroke.^3^ Unmet rehabilitation needs were more often reported by older adults and most often in the very old (>85 years). Depression, pain and poor-perceived general health were more common in those with unmet rehabilitation needs.

With regard to caregivers specifically, long-term follow-up conducted in 2016 – at three years (2011 cohort) and five years (2013 cohort) – documented outcomes in 5 053 informal stroke caregivers. In those supporting completely dependent survivors, less than half (49%) received support in their caregiving role at 3–5 years, and 24% expressed an unmet need of support. In caregivers for survivors with partial dependency, the proportion receiving community support was only 5%. Unsurprisingly, the degree of caregiver feelings of fatigue, frustration, anxiety and sadness increased with increasing degree of survivor dependency.^4^

Other European countries have national datasets relevant to life after stroke; however, few are as well established or maintained as in Sweden, and few follow up survivors longer term. With this caveat, some have produced useful data and have the potential to grow.

In Denmark, the stroke register currently only collects three-month follow-up data based on the modified Rankin Scale (mRs). However, the aim is to include one year follow-up data. ^5^

In Scotland, the focus of the national Scottish Stroke Care Audit (SSCA) has, to date, been acute care. This is not because longer term issues are perceived as unimportant but rather a lack of compelling evidence around life after stroke to inform measurable audit standards. This catch 22 clearly underlines the need for more research to provide such data and may be a good example of why other countries have not made progress. The Scottish system also gives an example of how routinely collected data can offer insights into life after stroke. Collection of patient-reported outcomes data at scale is challenging and resource intensive. The SSCA uses data on death, hospital stays and care-home admissions to calculate time spent at home over one year.^6^ This metric captures an outcome important for stroke survivors without the need for additional data collection, and the audit team are currently working on questions and indicators to move this forward.

In Italy**,** the Italian Project on Stroke at Young Age (IPSYS) is a nationwide registry that aims to collect data on those having a stroke as young adults (from 18 to 45 years). These data have been collected since January 2006. Although the registry is primarily focused on the acute phase of stroke, with a focus on identifying risk factors and improving medical management, it holds substantial data from long-term follow-up.^7, 8^ These studies will help to identify modifiable risk factors for stroke recurrence in a young population after their first stroke.

Aside from country-specific data collections, there are some excellent examples of regional and local longer term data collection.

In the UK, examples include the Oxford Vascular cohort^9^ and the South London Stroke Register (SLSR).^10^ SLSR is a community-based register of incident stroke patients registered continuously in one area of London since 1995. Several influential epidemiological studies have been reported from analysis of these data, and the focus most recently has been on charting the natural history of depression up to 24 years after stroke, reporting that the prevalence of depression was approximately 30% up to 15 years after stroke and increasing to 40% thereafter.^11^ Other research from the register has highlighted sex-related disparities in long-term outcomes after stroke, which suggest that women had significantly poorer outcomes in activities of daily living and more anxiety than men, with the sex differences persisting up to five years after stroke.^12^

In Germany, the Erlangen Stroke Project, a prospective, population-based stroke register started standardised data collection in 1994. A 20-year follow-up analysis found that nearly every second patient died five years after the first event.^13^ However, case-fatality rates decreased over the 20 years of observation, and the authors concluded that this decrease might be the result of improvements in stroke management and treatment in Germany. This data collection and follow-up has therefore clearly been important in monitoring stroke care across the pathway.

## *References*

1. Riksstroke. The Swedish Stroke Register, <https://www.riksstroke.org>.

2. Ullberg T, Zia E, Petersson J, et al. Changes in Functional Outcome Over the First Year After Stroke. *Stroke* 2015; 46: 389-394. DOI: doi:10.1161/STROKEAHA.114.006538.

3. Ullberg T, Zia E, Petersson J, et al. Perceived Unmet Rehabilitation Needs 1 Year After Stroke. *Stroke* 2016; 47: 539-541. DOI: doi:10.1161/STROKEAHA.115.011670.

4. Sennfält S and Ullberg T. Informal caregivers in stroke: Life impact, support, and psychological well-being—A Swedish Stroke Register (Riksstroke) study. *International Journal of Stroke* 2020; 15: 197-205. DOI: 10.1177/1747493019858776.

5. Kvalitetsinstitut S. Danks Stroke Register (DanStroke), <https://www.sundk.dk/kliniske-kvalitetsdatabaser/dansk-stroke-register/>.

6. McDermid I, Barber M, Dennis M, et al. Home-Time Is a Feasible and Valid Stroke Outcome Measure in National Datasets. *Stroke* 2019; 50: 1282-1285. DOI: doi:10.1161/STROKEAHA.118.023916.

7. Pezzini A, Grassi M, Lodigiani C, et al. Predictors of Long-Term Recurrent Vascular Events After Ischemic Stroke at Young Age. *Circulation* 2014; 129: 1668-1676. DOI: doi:10.1161/CIRCULATIONAHA.113.005663.

8. Bonacina S, Grassi M, Zedde M, et al. Long-term outcome of cervical artery dissection. *Neurological Sciences* 2020; 41: 3265-3272. DOI: 10.1007/s10072-020-04464-9.

9. Nuffield Department of Clinical Neurosciences - Medical Sciences Division. Oxford Vascular Study, <https://www.ndcn.ox.ac.uk/research/oxvasc>.

10. NHS Health Research Authority. South London Stroke Register, <https://www.hra.nhs.uk/planning-and-improving-research/application-summaries/research-summaries/south-london-stroke-register/>.

11. Lu Liu RP, Ajay Bhalla, Iain Marshall, Charles Wolfe, Matt O'Connell, Yanzhong Wang. NATURAL HISTORY OF DEPRESSION UP TO 24 YEARS AFTER STROKE: THE SOUTH LONDON STROKE REGISTER.

12. Xu M, Amarilla Vallejo A, Cantalapiedra Calvete C, et al. Stroke Outcomes in Women: A Population-Based Cohort Study. *Stroke* 2022; 53: 3072-3081. DOI: doi:10.1161/STROKEAHA.121.037829.

13. Rücker V, Heuschmann PU, O’Flaherty M, et al. Twenty-Year Time Trends in Long-Term Case-Fatality and Recurrence Rates After Ischemic Stroke Stratified by Etiology. *Stroke* 2020; 51: 2778-2785. DOI: doi:10.1161/STROKEAHA.120.029972.
