# Supplementary material for: Stroke Action Plan for Europe 2018–2030 (SAP-E): mid-term review and update
Source: Eur Stroke J. 2026 Jan 19;11(1):aakaf026. doi: 10.1093/esj/aakaf026 (PMC12866651; doi:10.1093/esj/aakaf026)
Supplement: aakaf026_Supplemental_Files [file aakaf026_supplemental_files.zip › Supplementary_file_3_aakaf026.docx]

# Supplementary file 3: Classification of outcome measures

| **Body structure (impairments)** | **Activities (limitations to activity = disability)** | **Participation (barriers to participation => handicap)** |
| --- | --- | --- |
| - Behavioural Inattention Test^a^ - Canadian Neurological Scale^b^ - Clock Drawing Test^c^ - Fugl-Meyer Assessment - Frenchay Aphasia Screening Test^d^ - General Health Questionnaire-28^e^ - Geriatric Depression Scale ^e^ - Hospital Anxiety and Depression Scale^e^ - Line Bisection Test^a,b^ - Mini-Mental State Examination^f^ - Modified Ashworth Scale^g^ - Montreal Cognitive Assessment ^f^ - National Institutes of Health Stroke Scale^b^ - Patient Health Questionnaire-9^e^ - Scandinavian Stroke Scale^b^ - Star Cancellation Test ^a^ - Stroke Aphasic Depression Questionnaire ^d^ - Western Aphasia Battery-Revised^d,h^ | - Action Research Arm Test - Barthel Index - Berg Balance Scale - Box and Block Test^h^ - Chedoke McMaster Stroke Assessment Scale^b^ - Fatigue Severity Scale^i^ - Frenchay Activities Index^b^ - Functional Ambulation Categories - Functional Independence Measure - Nine-hole Peg Test^h^ - Rivermead Mobility Scale^b^ - Six Minute Walk Test - Ten Meter Walk Test - Timed ‘Up-and-Go’ - Visual Analog Fatigue Scale^i^ | - Canadian Occupational - EQ-5D-5L - EuroQol Quality of Life Scale - London Handicap Scale - Medical Outcomes Study Short- Form 36 - Nottingham Health Profile - Performance Measure - Stroke Impact Scale^b^ - Stroke Specific Quality of Life^b^ |

Note: All of the above tools can be used to evaluate outcomes in stroke patients, but only stroke scales^b^ were developed to specifically assess stroke (neurological deficit) severity, while the rest are applicable to various neurological conditions with brain lesions.

^a^Tools developed to detect the presence of unilateral spatial inattention (neglect).

^b^Tools developed to specifically assess stroke (neurological deficit) severity.

^c^Tool used to quickly assess visuospatial and praxis abilities and that may determine the presence of attention and executive dysfunctions.

^d^Quick and simple tools to identify the presence of a language deficit.

^e^Tools developed to detect mood disorders after stroke [(e.g.](https://strokengine.ca/en/glossary/depression/) depression and anxiety).

^f^Rapidscreening instruments for detection and quantitative evaluation of cognitive impairment.

^g^Primary clinical measure of muscle spasticity in patients with neurological conditions.

^h^Paid clinimetric tools.

^i^Measures to assess post-stroke fatigue.
