# Supplementary material for: Stroke Action Plan for Europe 2018–2030 (SAP-E): mid-term review and update
Source: Eur Stroke J. 2026 Jan 19;11(1):aakaf026. doi: 10.1093/esj/aakaf026 (PMC12866651; doi:10.1093/esj/aakaf026)
Supplement: aakaf026_Supplemental_Files [file aakaf026_supplemental_files.zip › Supplementary_file_4_aakaf026.docx]

# Supplementary file 4: Key translational research topics

## Stroke prevention – improving vascular brain health

Vascular brain health – a central component of overall brain health – is essential for healthy ageing. Covert cerebral small vessel disease (SVD) as a manifestation of worse vascular health is detectable on brain imaging and highly prevalent in the ageing population. SVD is associated with a considerably increased risk of stroke and dementia and should be a major target to prevent these disabling conditions and promote healthier brain ageing. Reducing the burden of vascular brain disease requires more efficient disease prevention, with earlier detection and better stratification to enable personalised interventions, as well as mechanism-based therapies applicable for other main causes of ischaemic and haemorrhagic stroke, including large vessel atherosclerosis and aneurysm formation.

### Omics driven discovery and functional exploration

Genomics has developed as a major tool to reveal the genetic determinants of neurovascular disease and is increasingly leveraged to accelerate therapeutic developments and improve risk prediction and stratification. Meta-analyses of genome-wide association studies (GWAS) on up to millions of study participants have led to the discovery of more than 150 genetic loci harbouring common genetic risk variants for stroke and related phenotypes, such as magnetic resonance imaging (MRI) markers of SVD.^1-5^ Next-generation sequencing approaches have transformed the identification of rare genetic variants that cause monogenic strokes. Their exploration in large population biobanks has led to a revisiting of the frequency and penetrance of disease-causing mutations and revealed a blurring distinction between monogenic and multifactorial stroke, with shared underlying genes, pathways and possibly therapeutic targets.^3, 6, 7^ Leveraging genomic data, Mendelian randomisation is now widely used to support causal relations of putative risk factors with disease, while polygenic risk scores perform increasingly well for risk prediction and stratification,^1, 8^ with expected applications for personalised prevention and treatment or predictive and prognostic enrichment of clinical trials.^9^ Genetic evidence for drug effects are retrospectively estimated to increase the success rate of clinical trials by greater than two-fold.^10^ More recently, ‘multi-omics’ approaches that link genomics to other high-throughput technologies (e.g. epigenomics, transcriptomics, proteomics and metabolomics) applied to different tissues – including at single-cell resolution – have created unprecedented opportunities to decipher molecular and cellular mechanisms underlying stroke and vascular brain disease.^11-14^ While there is growing evidence that this advanced knowledge, combined with drug and compound databases, has the potential to accelerate drug discovery for stroke,^1^ the trajectory from discovery of genetic associations to therapeutic development remains complex.^15^

Deciphering precise pathways and mechanisms and testing therapeutic strategies ultimately requires complementing omics approaches with well-designed experimental models, such as gene silencing or gene overexpression in cellular and animal models.^16^ Massively parallel reporter assays are increasingly popular to allow larger scale screening of putative causal variants and genes among disease-associated genetic risk loci.^17^ Brain organoids, which enable recapitulatation of some level of brain structure and function with human-induced pluripotent stem cell lines in three-dimensional models, are an increasingly popular tool to screen for gene function and accelerate translation towards therapeutic developments, especially when combined with clustered regularly interspaced short palindromic repeats (CRISPR)-mediated gene editing and single-cell transcriptomic readouts.^18^ However, their application to vascular brain disease is challenging, and organ-on-a-chip models with microfluidic flow that mimic blood-brain-barrier (BBB) function and enable compound screening are an interesting alternative.

### Immune mechanisms and inflammation

Important insights into the role of immune mechanisms in the pathophysiology of atherosclerotic stroke, SVD and atrial fibrillation (AF) have emerged.^19, 20^ Atherosclerotic stroke is associated with a higher risk of early recurrence compared to other stroke aetiologies.^21, 22^ Atherosclerosis is a chronic, maladaptive inflammatory disorder.^19^ Cardiovascular risk factors, especially dyslipidaemia, activate the vascular endothelium and promote expression of adhesion molecules and inflammatory cytokines, which, in turn, recruit leukocytes and represent candidate targets for pharmacological interventions.^22, 23^ The post-stroke immune response accelerates existing plaque development and increases the risk of recurrent stroke.^24^ In addition, inflammatory markers such as C-reactive protein (CRP) and interleukin (IL) 6 have been associated with the occurrence of SVD,^25^ and CRP has been linked to the progression of SVD in stroke-free subjects.^26^ Systemic inflammatory triggers attributable to obesity, hypertension and viral infections may promote the development of AF.^27^ In population-based studies, CRP is associated with the occurrence of AF and the incidence of stroke in patients with established AF.^28, 29^ Elevated IL-6 levels have been associated with an increased risk of stroke in patients with AF and in the general population.^30, 31^ Clonal haematopoiesis of undetermined potential is independently associated with a two-fold increased risk of cardiovascular events^32^ and is thought to increase vascular risk by inducing monocytes to release excessive inflammatory cytokines.^33^ Initial studies on the therapeutic reduction of residual inflammatory risk in high-risk patients are underway, using colchicine, monoclonal antibodies and recombinant DNase as therapeutic agents.

## Acute stroke – reperfusion injury and neuroprotection

Current optimal treatment for acute ischaemic stroke is recanalisation with intravenous thrombolysis (IVT), endovascular thrombectomy, or both. However, full reperfusion is not achieved in many cases. In addition, not all patients benefit from endovascular treatment or are eligible for reperfusion therapies.

### Improving vessels patency and tissue reperfusion

Strategies designed to improve tissue reperfusion rates by dissolving distal microthrombi or to prevent thrombus formation locally in the capillary bed should be investigated – either alone or in combination with mechanical recanalisation. Recent research has proposed new molecules, as well as cellular and molecular targets, that may help achieve optimal reperfusion according to clot composition and location or by preventing thromboinflammation.

### Reducing reperfusion injury

Secondary complications of recanalisation and reperfusion therapies, such as haemorrhagic transformation, should be minimised. Complications due to treatments such as antiplatelet drugs and anticoagulants and the effective use of antidotes require further investigation. Current experimental evidence suggests that oxidative or nitrosative stress and thromboinflammation^34^ impair the benefits of reperfusion, with clinical investigations ongoing.

### Neuroprotection

Evaluation of neuroprotection therapies to prevent damage after reperfusion injury following recanalisation offers opportunities for novel and known drugs that have previously failed when administered too late or in the absence of reperfusion.^35^ Additional efforts are needed to identify effective approaches for late-phase protection.^36^ Targeting neuroinflammation or oedema may be key to identify neuroprotective mechanisms.^37, 38^ Attempts to alleviate progressive white matter changes remain under-represented in most animal models of stroke.

### Vascular protection

Protecting the vasculature, including the BBB, is critical in ischaemia and reperfusion to limit parenchymal injury. Microvascular dysfunction represents both a cause and consequence of ischaemic stroke and thereby is a potential therapeutic target for future drug development. Brain ischaemia can cause loss of myogenic tone and endothelial dysfunction that can lead to loss of autoregulation, sustained hypoperfusion and brain oedema leading to progression of cell death in the perilesional penumbra area.^39^ The efficacy of endovascular thrombectomy in ischaemic stroke opens novel avenues to reconsider the use of neurovascular protectants by hyperacute intra-arterial application, which have previously failed to reach efficacy by systemic administration in a broader patient population and deserve further testing.^40, 41^ Similarly, re-evaluation of other previously promising and heavily investigated compounds, including NXY-059 and tirilazad, in preclinical studies are warranted.^42^ Such studies may also promote the mechanistic understanding of no-reflow. Targeting brain perfusion in the late phase may prove useful,^43^ although effective modulation of cerebral perfusion in preclinical studies has been challenged by the risk of unwarranted blood flow redistribution and perfusion deficits.

### Glial protection

Like other cell types, astrocytes and microglia are severely affected by stroke. Glial injury leads to impaired regulation of ion gradients, augments excitotoxicity and pathological network activity, and contributes to brain oedema, BBB injury, inflammation and neuronal loss. Understanding the mechanisms of glial dysfunction after stroke is therefore of utmost importance.

### Inflammatory and immune responses

Promising cellular and molecular inflammatory mechanisms, with potential impacts on acute outcomes and post-stroke recovery, have been identified.^44^ Substantial basic research is required for more comprehensive understanding of the complex neuroimmune interactions before translational strategies are attempted. Central inflammatory states in the brain after stroke are linked with altered activity and dysfunction of glial cells that have emerging housekeeping roles.^45^ Stroke also triggers a multiphasic systemic inflammatory response that involves neural signals and soluble mediators, including cytokines, vasoactive substances, alarmins and acute phase proteins, followed by inhibition of immune responses that increases the risk of post-stroke infection. Imbalance of peripheral immune homeostasis after stroke represents a promising therapeutic target that requires investigation. Repurposing of available drugs in clinical use for other inflammatory conditions could improve the translational efficacy in testing anti-inflammatory therapies for stroke.^46^

## Rehabilitation

Expanding our knowledge of functional recovery mechanisms and potential therapeutic targets is key objective to enhance the effects of physical and cognitive rehabilitation in the chronic phase after stroke. Research is needed on neuronal plasticity and network recovery, and their interaction with delayed pathophysiological mechanisms such as neuroinflammation, apoptosis, neurogenesis and angiogenesis. This will require long-term follow-up in preclinical and clinical studies and integrating methods from stem-cell research, gene therapy, optogenetics, non-invasive brain stimulation and other fields. Consensus should be reached on the methods to identify functional recovery and neuroplasticity in experimental stroke models and stroke survivors.^47^

## Translational research and preclinical models

Numerous experimental therapies targeting thrombolysis, neuronal death and inflammation have been identified in animal models. However, their translation to clinical applications has mostly proven unsuccessful and few therapeutic approaches demonstrating efficacy in experimental stroke models have progressed to clinical trials, creating a pervasive ‘translational roadblock’. This barrier is often attributed to inherent weaknesses in preclinical studies.

Addressing this challenge requires preclinical studies to be conducted under conditions resembling those of real-life patients. This approach aims to enhance the likelihood of success for therapeutic candidates in treating ischaemic stroke. It involves using thromboembolic and thrombotic stroke models that faithfully reflect clinical settings, including clot composition and responsiveness to gold-standard treatments. Currently, experimental stroke models predominantly involve anesthetised animals, with anaesthesia regimens targeting N-methyl-D-aspartate (NMDA) and gamma-aminobutyric acid (GABA) receptors, thereby interfering with disease pathophysiology and response to treatments.

A promising alternative is the use of awake animals, with appropriate ethical considerations applied. In addition, stroke models need to be compatible with multimodal recording and imaging modalities such as Doppler/Doppler speckle or MRI. This ensures that readouts mirror the clinical situation. Whenever possible, incorporating non-human primates into stroke models, at least for safety assessments, holds significance.

In light of these considerations, it is recommended to conduct multicentric preclinical studies to validate drug candidates before their translation to clinical settings.^48, 49^ This collaborative approach can enhance the robustness and generalisability of findings, paving the way for more successful clinical applications in stroke research.

## References

1. Mishra A, Malik R, Hachiya T, et al. Stroke genetics informs drug discovery and risk prediction across ancestries. *Nature* 2022; 611: 115-123. DOI: 10.1038/s41586-022-05165-3.

2. Dichgans M, Pulit SL and Rosand J. Stroke genetics: discovery, biology, and clinical applications. *The Lancet Neurology* 2019; 18: 587-599. DOI: 10.1016/S1474-4422(19)30043-2.

3. Debette S and Markus HS. Stroke Genetics: Discovery, Insight Into Mechanisms, and Clinical Perspectives. *Circulation Research* 2022; 130: 1095-1111. DOI: doi:10.1161/CIRCRESAHA.122.319950.

4. Bordes C, Sargurupremraj M, Mishra A, et al. Genetics of common cerebral small vessel disease. *Nature Reviews Neurology* 2022; 18: 84-101. DOI: 10.1038/s41582-021-00592-8.

5. Bakker MK, van der Spek RAA, van Rheenen W, et al. Genome-wide association study of intracranial aneurysms identifies 17 risk loci and genetic overlap with clinical risk factors. *Nature Genetics* 2020; 52: 1303-1313. DOI: 10.1038/s41588-020-00725-7.

6. Grami N, Chong M, Lali R, et al. Global Assessment of Mendelian Stroke Genetic Prevalence in 101 635 Individuals From 7 Ethnic Groups. *Stroke* 2020; 51: 1290-1293. DOI: doi:10.1161/STROKEAHA.119.028840.

7. Cho BPH, Harshfield EL, Al-Thani M, et al. Association of Vascular Risk Factors and Genetic Factors With Penetrance of Variants Causing Monogenic Stroke. *JAMA Neurology* 2022; 79: 1303-1311. DOI: 10.1001/jamaneurol.2022.3832.

8. Myserlis EP, Georgakis MK, Demel SL, et al. A Genomic Risk Score Identifies Individuals at High Risk for Intracerebral Hemorrhage. *Stroke* 2023; 54: 973-982. DOI: doi:10.1161/STROKEAHA.122.041701.

9. Fahed AC, Philippakis AA and Khera AV. The potential of polygenic scores to improve cost and efficiency of clinical trials. *Nature Communications* 2022; 13: 2922. DOI: 10.1038/s41467-022-30675-z.

10. King EA, Davis JW and Degner JF. Are drug targets with genetic support twice as likely to be approved? Revised estimates of the impact of genetic support for drug mechanisms on the probability of drug approval. *PLOS Genetics* 2019; 15: e1008489. DOI: 10.1371/journal.pgen.1008489.

11. Montaner J, Ramiro L, Simats A, et al. Multilevel omics for the discovery of biomarkers and therapeutic targets for stroke. *Nature Reviews Neurology* 2020; 16: 247-264. DOI: 10.1038/s41582-020-0350-6.

12. Sliz E, Shin J, Ahmad S, et al. Circulating Metabolome and White Matter Hyperintensities in Women and Men. *Circulation* 2022; 145: 1040-1052. DOI: doi:10.1161/CIRCULATIONAHA.121.056892.

13. Yang Y, Knol MJ, Wang R, et al. Epigenetic and integrative cross-omics analyses of cerebral white matter hyperintensities on MRI. *Brain* 2022; 146: 492-506. DOI: 10.1093/brain/awac290.

14. Kuipers S, Overmars LM, van Es B, et al. A cluster of blood-based protein biomarkers reflecting coagulation relates to the burden of cerebral small vessel disease. *Journal of Cerebral Blood Flow & Metabolism* 2022; 42: 1282-1293. DOI: 10.1177/0271678x221077339.

15. Trajanoska K, Bhérer C, Taliun D, et al. From target discovery to clinical drug development with human genetics. *Nature* 2023; 620: 737-745. DOI: 10.1038/s41586-023-06388-8.

16. Asare Y, Campbell-James TA, Bokov Y, et al. Histone Deacetylase 9 Activates IKK to Regulate Atherosclerotic Plaque Vulnerability. *Circulation Research* 2020; 127: 811-823. DOI: doi:10.1161/CIRCRESAHA.120.316743.

17. McAfee JC, Bell JL, Krupa O, et al. Focus on your locus with a massively parallel reporter assay. *Journal of Neurodevelopmental Disorders* 2022; 14: 50. DOI: 10.1186/s11689-022-09461-x.

18. Paulsen B, Velasco S, Kedaigle AJ, et al. Autism genes converge on asynchronous development of shared neuron classes. *Nature* 2022; 602: 268-273. DOI: 10.1038/s41586-021-04358-6.

19. Libby P. Inflammation in atherosclerosis. *Nature* 2002; 420: 868-874. DOI: 10.1038/nature01323.

20. Cao J, Roth S, Zhang S, et al. DNA-sensing inflammasomes cause recurrent atherosclerotic stroke. *Nature* 2024; 633: 433-441. DOI: 10.1038/s41586-024-07803-4.

21. Lovett JK, Coull AJ and Rothwell PM. Early risk of recurrence by subtype of ischemic stroke in population-based incidence studies. *Neurology* 2004; 62: 569-573. DOI: doi:10.1212/01.WNL.0000110311.09970.83.

22. Georgakis MK, Bernhagen J, Heitman LH, et al. Targeting the CCL2–CCR2 axis for atheroprotection. *European Heart Journal* 2022; 43: 1799-1808. DOI: 10.1093/eurheartj/ehac094.

23. Cybulsky MI, Iiyama K, Li H, et al. A major role for VCAM-1, but not ICAM-1, in early atherosclerosis. *The Journal of Clinical Investigation* 2001; 107: 1255-1262. DOI: 10.1172/JCI11871.

24. Roth S, Singh V, Tiedt S, et al. Brain-released alarmins and stress response synergize in accelerating atherosclerosis progression after stroke. *Science Translational Medicine* 2018; 10: eaao1313. DOI: doi:10.1126/scitranslmed.aao1313.

25. Hoshi T, Kitagawa K, Yamagami H, et al. Relations of Serum High-Sensitivity C-Reactive Protein and Interleukin-6 Levels With Silent Brain Infarction. *Stroke* 2005; 36: 768-772. DOI: doi:10.1161/01.STR.0000158915.28329.51.

26. Walker KA, Windham BG, Power MC, et al. The association of mid-to late-life systemic inflammation with white matter structure in older adults: The Atherosclerosis Risk in Communities Study. *Neurobiology of Aging* 2018; 68: 26-33. DOI: <https://doi.org/10.1016/j.neurobiolaging.2018.03.031>.

27. Hu Y-F, Chen Y-J, Lin Y-J, et al. Inflammation and the pathogenesis of atrial fibrillation. *Nature Reviews Cardiology* 2015; 12: 230-243. DOI: 10.1038/nrcardio.2015.2.

28. Aviles RJ, Martin DO, Apperson-Hansen C, et al. Inflammation as a Risk Factor for Atrial Fibrillation. *Circulation* 2003; 108: 3006-3010. DOI: doi:10.1161/01.CIR.0000103131.70301.4F.

29. Lip GYH, Patel JV, Hughes E, et al. High-Sensitivity C-Reactive Protein and Soluble CD40 Ligand as Indices of Inflammation and Platelet Activation in 880 Patients With Nonvalvular Atrial Fibrillation. *Stroke* 2007; 38: 1229-1237. DOI: doi:10.1161/01.STR.0000260090.90508.3e.

30. Aulin J, Siegbahn A, Hijazi Z, et al. Interleukin-6 and C-reactive protein and risk for death and cardiovascular events in patients with atrial fibrillation. *American Heart Journal* 2015; 170: 1151-1160. DOI: <https://doi.org/10.1016/j.ahj.2015.09.018>.

31. Papadopoulos A, Palaiopanos K, Björkbacka H, et al. Circulating Interleukin-6 Levels and Incident Ischemic Stroke. *Neurology* 2022; 98: e1002-e1012. DOI: doi:10.1212/WNL.0000000000013274.

32. Jaiswal S and Libby P. Clonal haematopoiesis: connecting ageing and inflammation in cardiovascular disease. *Nature Reviews Cardiology* 2020; 17: 137-144. DOI: 10.1038/s41569-019-0247-5.

33. Belizaire R, Wong WJ, Robinette ML, et al. Clonal haematopoiesis and dysregulation of the immune system. *Nature Reviews Immunology* 2023; 23: 595-610. DOI: 10.1038/s41577-023-00843-3.

34. De Meyer SF, Denorme F, Langhauser F, et al. Thromboinflammation in Stroke Brain Damage. *Stroke* 2016; 47: 1165-1172. DOI: doi:10.1161/STROKEAHA.115.011238.

35. Savitz SI, Baron J-C, Yenari MA, et al. Reconsidering Neuroprotection in the Reperfusion Era. *Stroke* 2017; 48: 3413-3419. DOI: doi:10.1161/STROKEAHA.117.017283.

36. Fisher M and Savitz SI. Pharmacological brain cytoprotection in acute ischaemic stroke — renewed hope in the reperfusion era. *Nature Reviews Neurology* 2022; 18: 193-202. DOI: 10.1038/s41582-021-00605-6.

37. Thorén M, Escudero-Martínez I, Andersson T, et al. Reperfusion by endovascular thrombectomy and early cerebral edema in anterior circulation stroke: Results from the SITS-International Stroke Thrombectomy Registry. *International Journal of Stroke* 2023; 18: 1193-1201. DOI: 10.1177/17474930231180451.

38. Chamorro Á, Lo EH, Renú A, et al. The future of neuroprotection in stroke. *Journal of Neurology, Neurosurgery &amp; Psychiatry* 2021; 92: 129-135. DOI: 10.1136/jnnp-2020-324283.

39. Palomares SM and Cipolla MJ. Vascular Protection Following Cerebral Ischemia and Reperfusion. *J Neurol Neurophysiol* 2011; 2011. DOI: 10.4172/2155-9562.s1-004.

40. Fladt J, Guo J, Specht JL, et al. Infarct Evolution on MR-DWI After Thrombectomy in Acute Stroke Patients Randomized to Nerinetide or Placebo. *Neurology* 2024; 102: e207976. DOI: doi:10.1212/WNL.0000000000207976.

41. Hill MD, Goyal M, Menon BK, et al. Efficacy and safety of nerinetide for the treatment of acute ischaemic stroke (ESCAPE-NA1): a multicentre, double-blind, randomised controlled trial. *The Lancet* 2020; 395: 878-887. DOI: 10.1016/S0140-6736(20)30258-0.

42. Haupt M, Gerner ST, Bähr M, et al. Quest for Quality in Translational Stroke Research—A New Dawn for Neuroprotection? *International Journal of Molecular Sciences* 2022; 23: 5381.

43. Wardlaw JM, Woodhouse LJ, Mhlanga II, et al. Isosorbide Mononitrate and Cilostazol Treatment in Patients With Symptomatic Cerebral Small Vessel Disease: The Lacunar Intervention Trial-2 (LACI-2) Randomized Clinical Trial. *JAMA Neurology* 2023; 80: 682-692. DOI: 10.1001/jamaneurol.2023.1526.

44. Anrather J and Iadecola C. Inflammation and Stroke: An Overview. *Neurotherapeutics* 2016; 13: 661-670. DOI: 10.1007/s13311-016-0483-x.

45. Cserép C, Pósfai B and Dénes Á. Shaping Neuronal Fate: Functional Heterogeneity of Direct Microglia-Neuron Interactions. *Neuron* 2021; 109: 222-240. DOI: 10.1016/j.neuron.2020.11.007.

46. Yu IC, Kuo P-C, Yen J-H, et al. A Combination of Three Repurposed Drugs Administered at Reperfusion as a Promising Therapy for Postischemic Brain Injury. *Translational Stroke Research* 2017; 8: 560-577. DOI: 10.1007/s12975-017-0543-5.

47. Edwards JD, Dominguez-Vargas AU, Rosso C, et al. A translational roadmap for transcranial magnetic and direct current stimulation in stroke rehabilitation: Consensus-based core recommendations from the third stroke recovery and rehabilitation roundtable. *Neurorehabilitation and Neural Repair* 2024; 38: 19-29. DOI: 10.1177/15459683231209136.

48. Llovera G, Hofmann K, Roth S, et al. Results of a preclinical randomized controlled multicenter trial (pRCT): Anti-CD49d treatment for acute brain ischemia. *Science Translational Medicine* 2015; 7: 299ra121-299ra121. DOI: doi:10.1126/scitranslmed.aaa9853.

49. Lyden PD, Diniz MA, Bosetti F, et al. A multi-laboratory preclinical trial in rodents to assess treatment candidates for acute ischemic stroke. *Science Translational Medicine* 2023; 15: eadg8656. DOI: doi:10.1126/scitranslmed.adg8656.
