# Supplementary material for: Stroke Action Plan for Europe 2018–2030 (SAP-E): mid-term review and update
Source: Eur Stroke J. 2026 Jan 19;11(1):aakaf026. doi: 10.1093/esj/aakaf026 (PMC12866651; doi:10.1093/esj/aakaf026)
Supplement: aakaf026_Supplemental_Files [file aakaf026_supplemental_files.zip › Supplementary_file_5_Top-5_priorities_aakaf026.docx]

Supplementary file 5

Summary of top-5 priorities

|  | **Research and development: top 5 priorities** | **Targets for 2030: top 5 priorities** |
| --- | --- | --- |
| **Primary Prevention** | 1.Research on population strategies of stroke prevention at a governmental level and how to measure their efficacy – health economic approach and sustainability of those programmes.  2.Evidence on precision lifestyle medicine and precision medicine in preventing stroke.  3.Evidence to assess the benefits and potential harms of screening for stroke and stroke subtypes/aetiologies and cardiovascular disease risk factors in diverse populations, considering various approaches such as systematic and opportunistic screening.  4.Evidence on the effectiveness of digital health approaches in improving adherence with primary prevention interventions and their outcomes in stroke prevention.  5.Research on psychosocial factors and mental health concerning the risk and outcome of stroke. | 1.Reducing the age- and sex-standardised incidence of stroke by more than 15% by 2030 compared with 2018 (this is updated from the previous target to reduce total number of strokes by 10%).  2.Fully implementing national strategies for multi-sectorial public health interventions promoting and facilitating a healthy lifestyle and risk factor control (KPI 3a).  3.Having key stroke risk factors – hypertension, dyslipidaemia, atrial fibrillation, and hyperglycaemia – detected to the highest proportion and having people with high risk factors controlled, aiming at 80% of persons in target levels.  4.Implementing plans to promote brain health plans, including a focus on stroke-specific risk factors across the life course (KPI 3b – new)  5.Implementing pathways for nationwide opportunistic screening strategies for key risk factors, including hypertension, dyslipidaemia, hyperglycaemia and AF (KPI 3c – new). |
| **Organisation of acute stroke services** | 1.What are the most relevant barriers to the implementation of evidence-based stroke care?  2.What is the health-economic impact of stroke and the return of investment in stroke care? Which are the most cost-effective concepts to improve organisation of stroke care in countries with limited resources?  3.What are the optimum numbers and ratios of stroke centres and stroke units per million population for municipal and rural areas?  4.What is the role of telemedicine systems for acute stroke, rehabilitation and long-term care?  5.What elements are needed to enable more effective participation in decision-making among patients and relatives? | 1.Implementing a national stroke plan that defines pathways, care and support after a stroke, including pre-hospital phase, hospital stay, discharge and transition, follow-up, and life after stroke. These pathways should involve the public and should be adaptable to regional circumstances to ensure equal access to stroke care, regardless of patient age, characteristics, region and time of hospitalisation (KPI 1).  2.Establishing a scientific stroke society and SSO in each country.  3.Having at least one individual from the respective SSO equally involved and supported during the development of each country’s national stroke plan and stroke-related guidelines (KPI 2).  4.Treating 90% or more of all patients with acute stroke in Europe in a stroke unit as the first level of care (KPI 6a).  5.Treating 90% or more of all patients with acute stroke in Europe in a stroke unit within 24 hours after admission to hospital as the first level of care (KPI 6b). |
| **Acute Management** | 1.How can the speed, safety and effectiveness of reperfusion approaches (drugs or devices) be optimised in Europe?  2.Which pharmacological or other strategies will reduce the extent of irreversible brain damage in ischaemic stroke patients before recanalisation therapies are started?  3.Which strategies will improve outcomes in ischaemic stroke patients who are not eligible for reperfusion therapies or who do not recover after recanalisation?  4.Which treatment strategies will improve outcomes in patients with ICH: haemostatic and surgical approaches, prevention of secondary injury, and intensive and tailored blood pressure management?  5.Which treatment strategies will further improve outcomes in patients with SAH by reducing brain injury? | 1.Achieving national IVT rates above 20% of all patients with ischaemic stroke (KPI 7a).  2.Achieving national MT rates above 7.5% of all patients with ischaemic stroke (KPI 7b).  3.Median door-to-needle time <30 minutes in MT (KPI 7c) and median door-to-groin <60 minutes in IVT (KPI 7d – new).  4.First-month case-fatality rates <15% for all stroke patients (KPI 13a – new).  5.First-month case-fatality rates <10% after ischaemic stroke (KPI 13b – new), first-month case-fatality rates <30% for ICH (KPI 13c – new), and first-month case-fatality rates <25% for SAH (KPI 13d – new) |
| **Secondary prevention and follow-up** | 1.Can access and adherence to secondary prevention be improved? (Specific attention to new technologies and approaches, as well as poorly represented underserved groups and long-term follow-up.)  2.Can secondary prevention be personalised (e.g. through biomarkers and genetic data)?  3.Can we identify specific interventions and approaches that reduce the progression of SVD and its clinical outcomes, including stroke and cognitive decline?  4.Have improvements in best medical therapy changed the threshold for carotid intervention?  5.What is the optimal treatment strategy in patients with AF and a significant risk factor for haemorrhagic stroke (e.g. previous haemorrhage or cerebral amyloid angiopathy)? | 1.Including secondary prevention in national stroke plans, with follow-up in primary/community care, and ensuring/stimulating translation into local protocols and guidelines.  2.Ensuring that initiation of basic secondary prevention is monitored in quality and outcome assessment programmes.  3.Ensuring that at least 90% of patients have access to basic secondary prevention, including antithrombotics, antihypertensives and statins, as well as lifestyle advice, and that this is monitored (KPI 10).  4.Ensure that at least 90% of the stroke population is seen at a 3–6-month post-stroke follow-up visit (KPI 12a); this can be done by the discharging stroke unit or the general practitioner.  5.Implementing a post-stroke checklist to follow up on secondary prevention, as well as other factors of life after stroke (KPI 12b). |
| **Rehabilitation** | 1.Developing evidence-based rehabilitation programmes based on timing, dosing, level, long-term duration and type of intervention.  2.Developing efficient management programmes for fatigue, anxiety and cognitive impairments after stroke.  3.Designing clinical trials defining how to reach maximal neurological potential in each stroke patient.  4.Documenting the potential benefit of maintenance training.  5.Developing a post-stroke rehabilitation guideline defining best practice rehabilitation. | 1.Providing early stroke unit rehabilitation in at least 90% of stroke units (KPI 9a).  2.Providing ESD in at least 60% of stroke units (KPI 9b) (from the stroke unit or from a community service).  3.Providing a documented individual plan for community rehabilitation and self-management support for all stroke patients with residual difficulties on discharge from hospital to at least 60% of patients (KPI 11).  4.Ensuring that all stroke patients and carers have a review of their rehabilitation and other needs at 3‒6 months after stroke and annually thereafter (KPI 12a and KPI 12b).  5.Involving and supporting stroke survivors and their carers during decision-making to ensure that they make informed decisions about their rehabilitation goals. |
| **Life after stroke** | 1.What are the experiences and needs of stroke survivors at different times during their lifespan, considering different cohorts of stroke survivors and challenges of those with multiple morbidities – and their carers – to inform the design of optimal care pathways.  2.What would a model of best care and long-term support look like? This should include the opportunity for reviews and specific roles to provide holistic coordinated support.  3.How can data on life after stroke best be collected within stroke registries to improve understanding of the long-term outcomes of stroke and service planning, and what data should this comprise?  4.What products and services (digital and physical) would support self-management, community integration, education and healthcare?  5.How can high-quality information and training to help non-specialist staff, especially social care staff, be targeted? It is envisaged that this will involve research around staffing levels, core competencies and the involvement of non-governmental and non-profit-making bodies such as charities and voluntary groups | 1.Providing comprehensive stroke follow-up that addresses all aspects relevant for life after stroke (KPI 12a).  2.Using a recognised post-stroke checklist and functional assessment to capture all stroke-related health problems. People should be referred on as appropriate (KPI 12b).  3.Providing equitable support, established through national stroke care plans and in conjunction with SSOs, to stroke survivors, regardless of their place of residence and socioeconomic status. Minimum standards should be agreed for what every stroke survivor should receive regardless of where they live (KPI 1 and KPI 2).  4.Ensuring appointment of government-level individuals or teams responsible for inclusion of life after stroke in national stroke plans, with supporting national databases in place for quality improvement.  5.Exploring implementation of supported self-management information and assistance systems needs as a priority area. |
| **Evaluation of outcomes and quality improvement** | 1.What definitions should be used across Europe for recording and reporting of data on stroke and TIA?  2.How can data on the quality of care be used to compare process and outcomes of care, taking into account variations in case-mix, and what is the minimum dataset that is needed?  3.What sustainable systems are needed to allow international comparisons of the clinical and cost-effectiveness of care and reporting of within-country variations and variations by other factors such as geographical region (urban versus rural) and over time?  4.Which strategies support the effective use of clinical guidelines and clinical quality registry data to inform health/stroke service delivery?  5.How can new technologies be used to (a) extract audit or register data automatically from electronic patient records to reduce burden on stroke services and increase consistency in data collection and (b) to conduct simultaneous data evaluation in multiple national and regional registers without the need to transfer large datasets and without data protection issues? | 1.Defining a common European framework of reference for stroke care quality, including:   - Strengthening the development of updated European guidelines for management of acute stroke care, longer term rehabilitation and prevention; where appropriate and sustainable, the living guideline model could be adopted. - Expanding and implementing the SST as the tool to enable accurate international comparisons of care at health system level in hospital and in the community (including structure, process, outcome measures and patient experience). - Assigning a named individual who is responsible for stroke quality improvement in each country or region.   2.Defining a common European framework of patient metrics and variables reflecting quality indicators, including:   - a minimal dataset that should be provided as a part of patient documentation - a data dictionary that defines quality indicators - a list of recommendations to ensure interoperability between different national and international registries.   3.Establishing national- and regional-level systems for assessing and accrediting stroke clinical services, providing peer support for quality improvement and making audit data available to the public (KPI 4).  4.Regular certification or equivalent auditing processes for quality improvement of all stroke units and other stroke services (KPI 5).  5.Collecting patient-reported and longer term outcomes (e.g. six months and one year), covering hospital and community care, considering digital health solutions for this purpose (e.g. web apps). |
| **Translational stroke research** |  | 1.Creating an organisational framework by implementing confirmatory preclinical research through ‘team science’ and by providing novel tools for advanced trial designs to increase validity.  2.Developing and implementing guidelines for preclinical stroke studies on new treatments to maximise the success of clinical translation.  3.Focusing experimental stroke research on identifying new treatable targets with high translational potential that will lead to successful clinical trials by 2030.  4.Identifying novel therapeutic targets for subtypes of stroke with no specific mechanistic treatment available to date, especially cerebral SVD and ICH. |
